# Elevated Toxic Element Emissions from Popular Disposable E‑Cigarettes: Sources, Life Cycle, and Health Risks

**DOI:** 10.1021/acscentsci.5c00641

**Published:** 2025-06-25

**Authors:** Mark R. Salazar, Lalima Saini, Tran B. Nguyen, Kent E. Pinkerton, Amy K. Madl, Austin M. Cole, Brett A. Poulin

**Affiliations:** † Department of Environmental Toxicology, 8789 University of California Davis, Davis, California 95616, United States; ‡ Center for Health and the Environment, 8789 University of California Davis, Davis, California 95616, United States; § Interdisciplinary Center for Plasma Mass Spectrometry, 8789 University of California Davis, Davis, California 95616, United States

## Abstract

The rapidly evolving market of disposable e-cigarettes poses unknown health risks to adolescents and young adults. We report excessive emissions of toxic metallic elements in aerosols from flavored and “clear” versions of three popular products (Esco Bar, Flum Pebble, and ELF Bar), orders of magnitude higher in concentration than traditional cigarettes and other e-cigarettes. Heating coil elements (chromium (Cr), nickel (Ni)) likely leached into e-liquids and aerosols from coil degradation during use, increasing up to 1000-fold in concentration over the device life. In Esco Bar devices, high concentrations of lead (Pb, ≤175 ppm), Ni (≤38 ppm), copper (Cu, ≤546 ppm), and zinc (Zn, ≤462 ppm) were observed in both e-liquids and aerosols. We identified the illicit use of leaded bronze in nonheating device components in contact with e-liquid as the source of Pb. Elevated antimony (Sb) in Flum Pebble and Esco Bar samples had unknown origins. Analyses showed Cr was present as nontoxic Cr­(III), while Sb was a mixture of nontoxic Sb­(V) and carcinogenic Sb­(III). Risk assessments revealed cancer risks from Ni and Sb­(III) and noncancer toxicity risks from Pb and Ni exceeded safety thresholds. These findings highlight critical gaps in e-cigarette regulation, characterization, and enforcement, with implications for public health.

## Graphical abstract

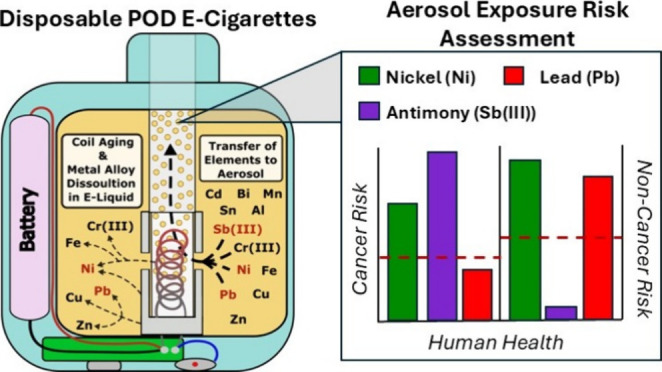

## Introduction

Disposable POD (dPOD) e-cigarettes, currently the most modern form of electronic nicotine delivery systems, have greatly expanded in popularity and are now the most popular e-cigarettes on the market, particularly among adolescent children.^1–3^ Despite their prevalence, research has been slow to catch up to market trends, with few studies on disposable e-cigarettes in the past three years since the introduction of these devices, leaving consumers and regulatory bodies unaware of associated risks.^4^ In contrast to combustible cigarettes, disposable e-cigarettes contain metal coils and other metallic components in continual contact with “e-liquid” solution (containing nicotine salt, flavorings, and organic acids) that are aerosolized and inhaled by humans. Nicotine salts introduce organic acids^5^ in e-liquids, which may facilitate metal dissolution in e-liquids through ligand–metal interactions.^6^ Recent studies measured metals and metalloids including nickel (Ni), chromium (Cr), antimony (Sb), and lead (Pb) in the aerosols of a limited number of dPODs tested,^7–9^ but to the best of our knowledge, no assessments have been conducted on ELF Bars, Flum Pebbles, and Esco Bar disposable e-cigarettes. The U.S. Centers for Disease Control and Prevention (CDC) National Youth Tobacco Survey (NYTS) identified ELF Bars as the most popular e-cigarette used by youth in the U.S. in both 2023 and 2024, while Esco Bars were the second most popular in 2023 and sixth in 2024.^2,3^ At present, nearly all disposable e-cigarettes are not authorized for sale in the U.S.^10–12^ The FDA has issued several warning letters and taken enforcement action against the manufacturers of ELF Bar and Esco Bar in its commitment to protect youth against illegal flavored, disposable e-cigarettes, yet these products still exist in the market and are among the most popular with youth.^2,3,13,14^

E-cigarette heating coils consist of primarily Cr, iron (Fe), and Ni often as alloys Nichrome (Ni–Cr), Kanthal (Al–Cr–Fe), and stainless steel (Cr–Fe–Ni),^15,16^ which have been attributed to increases in metal and metalloid emissions with device use possibly due to coil degradation or leaching.^15,17–21^ Respiratory exposure to these elements at sufficient doses can increase the risk of potential serious health effects such as neurotoxicity,^22–24^ cardiovascular disease,^25^ renal disease,^23^ respiratory disease,^26^ and lung cancer,^27^ with children being disproportionately susceptible to the neurotoxic effects of Pb.^23,29^ Redox-active elements (e.g., Fe) present in aerosols may facilitate production of excess reactive oxygen species (ROS),^5,28,30^ which can damage cells and induce inflammation, contributing to the development of respiratory diseases such as cancer, asthma, and lung fibrosis.^31,32^ A recent study exposed mice to aerosols from menthol flavored JUUL e-cigarettes that contained Fe and to house-dust mite allergen, reporting changes in gene expression for Mmp12, markers for oxidative stress, iron metabolism, inflammation and immune defense; though the changes were not always conclusive.^33^ E-cigarette use by adolescent children and young adults has been shown to result in elevated concentrations of metals and metalloids in blood, urine, and saliva^34–37^ with unknown implications for cancer and noncancer risk outcomes. Yet, important knowledge gaps remain on dPOD e-cigarettes, including studies on the most popular brands on the market, an understanding of where metals and metalloids in the aerosols may originate, mechanisms of metal and metalloid release over the device life cycle, and the oxidation state (and thus toxicological risk) of Cr and Sb.^23,38–40^ Risk assessments over a lifetime of dPOD use are needed to accurately predict the potential health outcomes.

This study combined complementary trace element analytical techniques to identify the elemental composition of metallic heating coils and other internal components that potentially contribute to metal and metalloid emissions in e-cigarette aerosols of three highly popular yet understudied brands of dPOD e-cigarettes. Heavily flavored and lightly flavored (termed “Clear”) devices were evaluated. Metal and metalloid concentrations were quantified in virgin e-liquids (i.e., prior to device use), aged e-liquids (i.e., after device use), and aerosols over the device life cycle. The oxidation states of Cr (nontoxic Cr­(III) vs carcinogenic Cr­(VI)) and Sb (carcinogenic Sb­(III) vs nontoxic Sb­(V)) were quantified in e-cigarette aerosols. Metal and metalloid concentration and oxidation state data were used to inform cancer and noncancer risk assessment analyses. Our findings highlight the unforeseen noncancer and cancer health risks posed by dPODs from exposure to Pb, Sb­(III), and Ni.

## Results and Discussion

### Elemental Composition of E-Cigarette Device Components

For each of the three brands of devices (Esco Bar, Flum Pebble, and ELF Bar, each in Flavored and Clear), we identified the elemental composition of all metallic components that contact e-liquids (wire coils, mesh coils, sheaths, wire coil coatings, and mesh coil supports) by laser ablation inductively coupled plasma mass spectrometry (LA-ICP-MS). Elemental abundance data of four devices are presented in Figure 1; photographs, diagrams of devices and device components, and tabulated data are presented in Figures S1–S9 and Table S1. The heating coils in all analyzed dPOD devices were made primarily of metal alloys containing Cr, Ni, and Fe of varying abundance (16% to 42%, 0% to 57%, and 1% to 83%, respectively; Figure 1, Table S1). ELF Bar wire coils had different alloy compositions based on the device type (Flavored, Clear, and 0% Nicotine), with ELF Bar Flavored and 0% Nicotine consisting of Nichrome-like composition (42% Cr, 54–57% Ni, ≤2% Fe; Figure 1A) and the ELF Bar Clear resembling Kanthal (24% Cr, 0.1% Ni, 75% Fe) (Figure 1B, Table S1).^16^ In contrast, Flum Pebble devices used mesh coils (in both Flavored and Clear devices) composed of stainless steel alloys of 53% Fe > 24% Cr > 10% Ni > 7% molybdenum (Mo) > 3% Cu > 2% manganese (Mn) > 1% cobalt (Co) (Figure 1C, Table S1).^16^ Esco Bar mesh coils (Flavored and Clear) consisted of 75% to 83% Fe, 16% to 24% Cr, and 0.5% Al consistent with Kanthal,^16^ similar to the ELF Bar Clear wire coil (Figures 1B and 1D, Table S1). Wire coil coatings and mesh coil supports, likely serving to maintain coil structure and conductivity (Figures S9C–S9H), were primarily composed of 83% to 99.8% Ni with the exception of the Flum Pebble Clear device (53% Fe, 24% Cr, 10% Ni, 7% Mo, 3% Cu, 2% Mn, and 1% Co) (Table S1).

**Figure 1:**
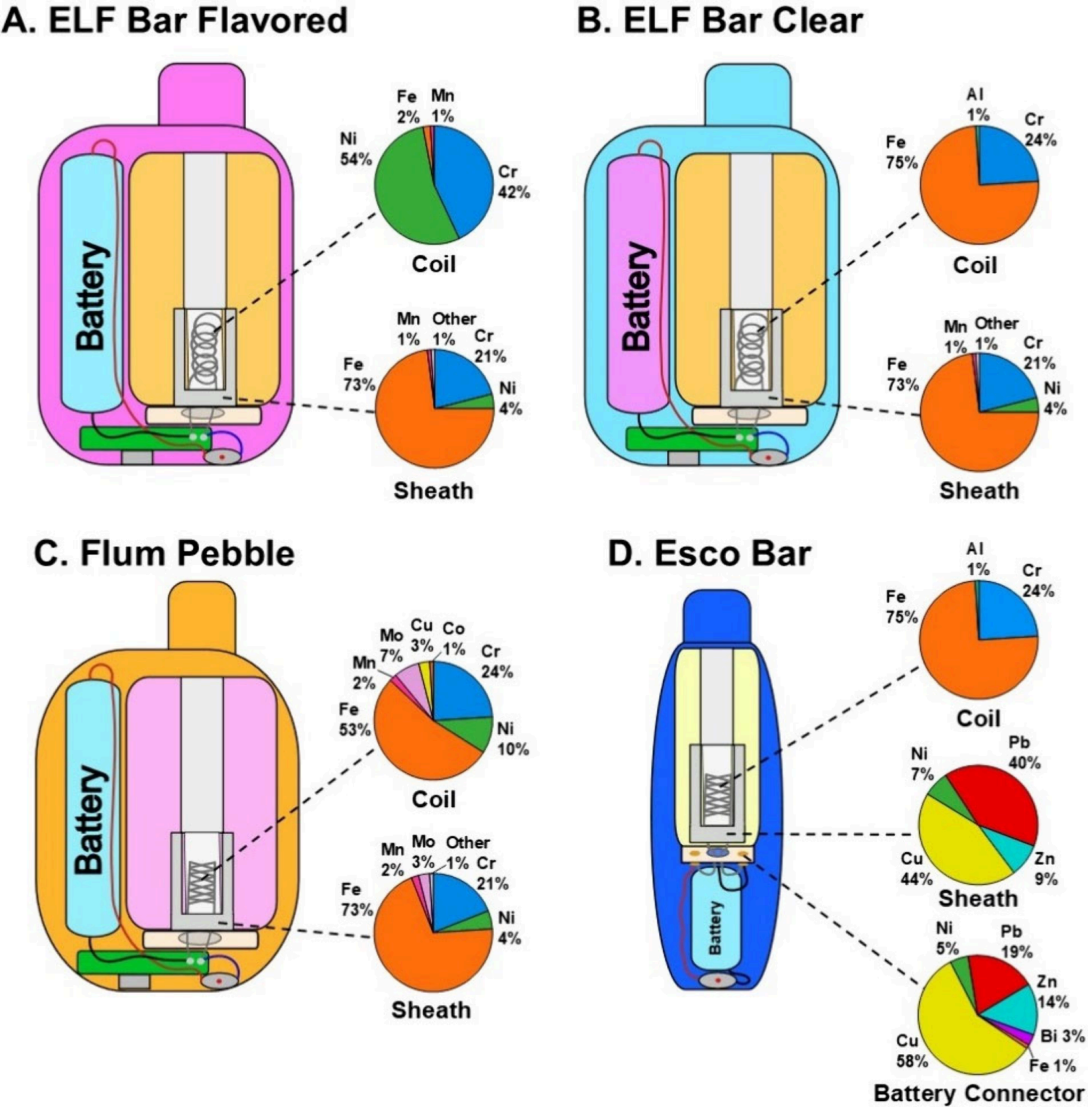
Elemental compositions of device coils, sheaths, and battery connectors of disposable e-cigarettes (dPODs) (elements >1% shown, elements <1% listed as “Other”): ELF Bar (Flavored (A) and Clear (B)), Flum Pebble (C), and Esco Bar (D). Elemental abundances reported as mean values (*n* = 3; Table S1). See Figure S2 for detailed identification of device internal components.

The composition of coil sheaths, which provide structure to the air flow pathway for aerosol delivery and presumably convey e-liquids to the coil (Figures 1, S1C, S2, S5D, and S6E), showed relatively high elemental compositions of Cu, Pb, Fe, and Cr. Esco Bar Flavored sheath consisted of 44% Cu, 39% Pb, 9% Zn, and 7% Ni (Figure 1D, Table S1), which is consistent with a leaded bronze alloy. ELF Bar and Flum Pebble sheaths, in contrast, were composed of 69% to 73% Fe, 19% to 21% Cr, 4% to 5% Ni, and 0% to 4% Mo (Figures 1A–1C, Table S1). Further, wires that connect to device batteries in Esco Bar devices (termed “battery connectors”; Figure S5) come into contact with e-liquids and were composed of 58% Cu, 19% Pb, 14% Zn, and 5% Ni (Table S1), sharing the same dominant metals as the Esco Bar sheath.

In brief, the distribution of metals and metalloids in heating coils (Cr, Fe, and Ni) were distinct from nonheating components of sheaths (Cu, Pb, Zn), wire coatings and mesh support (Ni), battery connectors (Cu, Pb, Zn), and wiring solder (Sn, Cu), which provides critical information to assess sources of elements identified in e-liquids and aerosols. Whether leaded bronze alloys were used in Esco Bar dPODs intentionally or otherwise, a material containing 20–40% Pb in contact with corrosive vape e-liquid poses a potential cause for concern and warrants further investigation on implications of aerosol exposures and health risks.

### Element Concentrations and Sources in Virgin E-Liquids

For all devices in this study, the concentrations of metals and metalloids were measured in virgin e-liquids (i.e., e-liquids prior to device operation), aerosol samples collected at 100 puff intervals to a maximum of 500–1500 puffs (until device expiration), and aged e-liquids (removed from devices after 500 puffs). The goals were to (1) assess the origins of metals and metalloids in dPOD aerosols (i.e., heating coils, nonheating device components, or virgin e-liquid materials) and (2) identify if device operation influenced element concentrations in aerosols. Across all devices, virgin e-liquids exhibited relatively low concentrations of the primary elements observed in the heating coils, including Cr (3 to 20 μg/kg) and Fe (148 to 1090 μg/kg) (Table S2). Across ELF Bar and Flum Pebble virgin e-liquids, Ni was similarly low to Cr and Fe (14 to 29 μg/kg; Table S2). Unexpectedly, elements that are not present in heating coils (Table S1), including Pb, Cu, Zn, and Sb, were observed at excessive concentrations in Esco Bar device virgin e-liquids, with the exception of Ni which was elevated in virgin e-liquids relative to ELF Bar and Flum Pebble virgin e-liquids and present in coils (Figure 1). Esco Bar Flavored and Clear virgin e-liquids showed extremely high concentrations of Pb (64,000 to 127,000 μg/kg), Ni (13,000 to 38,400 μg/kg), Cu (344,000 to 533,000 μg/kg), and Zn (240,000 to 376,000 μg/kg) (Table S2). For context, concentrations of Pb, Ni, Cu, and Zn were universally and comparatively low in all other virgin e-liquids from Elf Bar or Flum Pebble devices, at ≤15, ≤29, ≤24, and ≤331 μg/kg, respectively, with the exception of Zn in the ELF Bar Flavored virgin e-liquid at 4420 μg/kg (Table S2). Sb concentrations in Flum Pebble e-liquids (Flavored and Clear) ranged from 2050 to 2380 μg/kg, and for Esco Bar devices and ELF Bar Flavored 0% Nicotine ranged from 510 to 2220 μg/kg, whereas other ELF Bar devices contained ≤7 μg/kg.

The source(s) of metals and metalloids measured in virgin e-liquids were assessed using the elemental composition of device components (Figure 1, Tables S1 and S2). In Esco Bar devices, the relatively high concentrations of Pb, Ni, Cu, and Zn in virgin e-liquids align with the composition of the leaded bronze alloy sheaths and battery connectors (Figure 1D). This evidence suggests that these two metallic components are the sources of these metals leaching into Esco Bar e-liquids prior to device operation. Sb was not identified in any internal metallic component analyzed but was present at comparatively high concentrations in select e-liquids (Flum Pebble, Esco Bar, and ELF Bar Flavored 0% Nicotine) (Table S2). Thus, in virgin e-liquids, metals and metalloids from heating components were at relatively low concentrations across all devices aside from Ni, whereas contamination of virgin e-liquids was observed due to leaching of elements from nonheating components (sheath and battery connectors releasing Pb, Cu, and Zn) or from unknown sources (Sb).

### Elements Emitted in Aerosols

Metal and metalloid concentrations were measured in aerosols of dPODs over device life cycles. A single device of ELF Bar Flavored, ELF Bar Clear, and Flum Pebble Flavored products were used to quantify changes in metal and metalloid concentrations over the complete device life cycle (1500 puffs ELF Bar Flavored, 1300 puffs ELF Bar Clear, 1400 puffs Flum Pebble Flavored) (Figures 2 and S10–S13, Table S3). An aging analysis to 1500 puffs was planned for an Esco Bar Flavored device, but the 2500 puff capacity Esco Bar devices lost power after 300 puffs and became inoperable between 400 and 500 puffs; therefore, the aging study was restricted to 500 puffs for these devices. In the three devices evaluated between 100 and 1500 puffs, the concentrations of Cr and Ni increased in aerosols with device age as defined by usage or puff number (Figures 2A and 2B). These are dominant elements present in heating coils (Figure 1); thus, we interpret that increases in Cr and Ni concentrations in aerosols with device age are due to the release of these elements from heating coils, as corroborated by coil composition analyses (Figure 1). For example, the concentrations of Cr and Ni in aerosols of ELF Bar Flavored and Flum Pebble Flavored devices increased 1.4 to 2.7 orders of magnitude over the device life cycle (Figures 2A and 2B). For the ELF Bar Flavored device, Cr and Ni increased from 4 to 1960 μg/kg and 37 to 19,000 μg/kg between 100 and 1500 puffs, respectively (Figures 2A and 2B). For the Flum Pebble Flavored device, concentrations of Cr and Ni increased from 100 to 1000 puffs and then decreased from 1000 to 1300 puffs (Figures 2A and 2B) consistent with reduced aerosol mass generation at the end of the device life cycle (Figure S10). The observed increase and subsequent decrease in Cr and Ni concentrations at the end of the device life cycle in the Flum Pebble Flavored device may be the result of an extensive coil degradation event at 1000 puffs releasing large amounts of elements primarily to the aerosol rather than to the e-liquid, which might explain why element concentrations decreased after 1000 puffs rather than increased (Figures 2A and 2B). An event of coil degradation could also explain the decrease in aerosol generation from 1000 to 1400 puffs (Figure S10).

**Figure 2:**
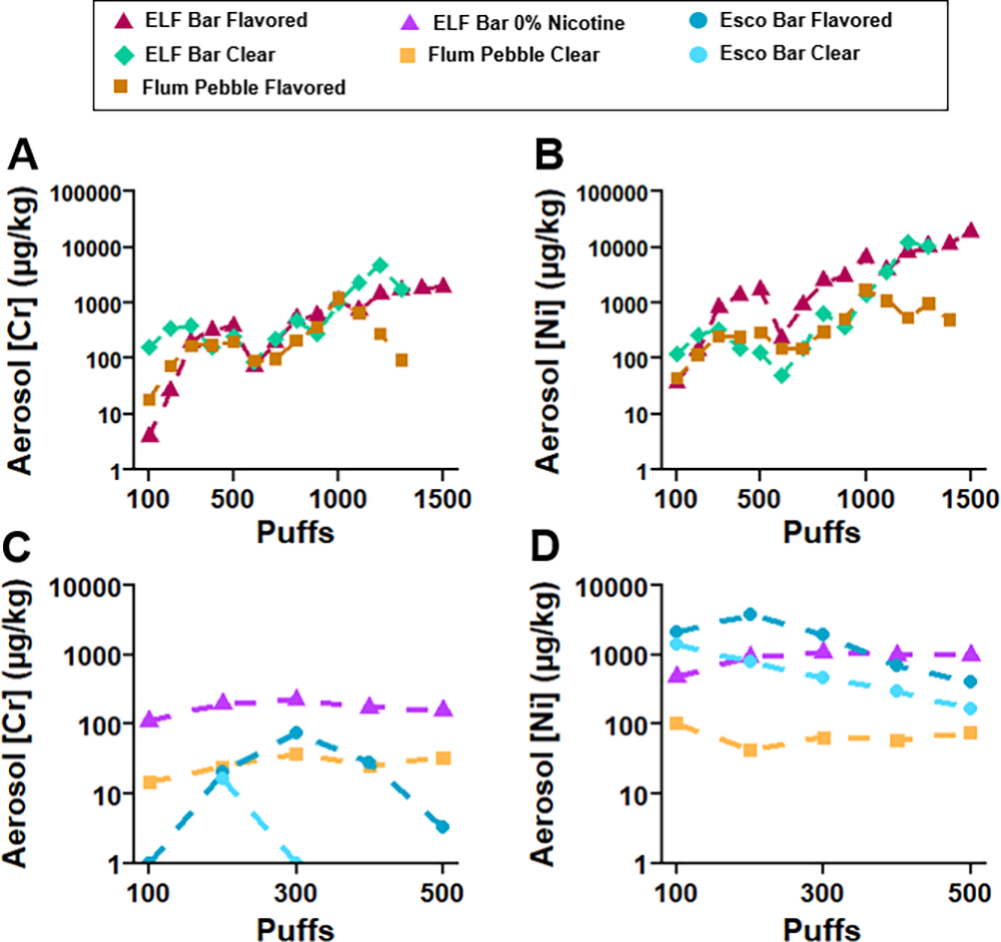
Aerosol concentrations (μg/kg) of total chromium (Cr) and nickel (Ni) for (A and B) the full aging analysis (100 to 1500 puffs) for three devices (ELF Bar Flavored and Clear, Flum Pebble Flavored) and (C and D) 100 to 500 puffs for four remaining devices (ELF Bar Flavored 0% Nicotine, Flum Pebble Clear, Esco Bars Flavored and Clear). See Figures S11–S13 for plots of all elements and Table S3 for all aerosol elemental concentrations. Element concentrations below the method limit of quantification (LOQ) are reported as estimates in Table S3.

For all seven devices in this study, element concentrations were measured in experimental triplicate to 500 puffs (Table S3), and similar increases in concentrations of Cr and Ni were observed with increased puff count (Figures 2C and 2D) as observed for the three devices evaluated to 1500 puffs (Figures 2A and 2B). The one exception was for Esco Bar devices, where Ni concentrations were highest at 100 puffs due to elevated concentrations of Ni in virgin e-liquids and decreased with increased puffs due to the loss of device power (Figure 2D). The linearity between mean aerosol element concentrations and puff count (to 300 and 500 puffs) was assessed, and coefficients of determination (*R* ^2^) are provided in Table S4. Linear increases (*R* ^2^ > 0.7) were observed for Cr and Ni across most devices from 100 to 300 puffs (Table S4). However, these increases were not conserved from 100 to 500 puffs with the exceptions of ELF Bar Flavored and Flum Pebble Flavored. This may suggest that metal leaching may occur at inconsistent time points in device life cycles across flavors and brands.

Significant differences were observed between Flum Pebble Flavored and Clear aerosols for Cr (*p* = 0.035) and Ni (*p* = 0.040) cumulative emissions from 100 to 500 puffs (Table S5.2). These elements were identified as primary constituents in Flum Pebble coils (Figure 1C), which may suggest flavoring-induced enhancement of Cr and Ni dissolution from the coil to the e-liquid. However, significant differences were not observed in the concentration of these elements between ELF Bar flavors and nicotine versus nicotine-free devices (Table S5.2).

To further confirm if increases in the concentration of Cr and Ni in aerosols with device use were likely due to these elements being released from heating coils to e-liquids, the residual aged e-liquids (after 500 puffs) were collected from devices, analyzed for element concentrations, and compared with virgin e-liquids (Table S2). Across all devices, aged e-liquids were significantly higher in Cr and Ni concentration compared to virgin e-liquids (paired *t* test; *p* < 0.001 to 0.045, Figure S14, Table S6). In the case of Cr, aged e-liquids were enriched, on average, 17-fold in concentration compared with virgin e-liquids across all seven devices (Table S2). A 1:1 linear correlation was observed between the concentrations of Cr and Ni in aged e-liquid and 500-puff aerosols (Table S7), confirming that the concentrations of these elements in e-liquids were directly proportional to aerosol concentrations due to transfer during device operation, consistent with observations from third generation MOD e-cigarettes.^18,19^ Differences in the degree to which Cr and Ni concentrations increased with device use in aged e-liquids and, thus, aerosols may be explained by variable alloy composition and coil type (i.e., wire or mesh coils) (Figure 1, Table S1). Taken together, Cr and Ni concentrations were relatively low in virgins e-liquids and increased with device age due to the likely release of these elements from coils during heat cycling, which has been observed for previous generation e-cigarette devices.^15,17–20^ These findings explain the systematic increases in Cr and Ni concentrations in aerosols over device life cycles (Figures 2A and 2B).

Several other metals and metalloids, including Pb, Cu, Zn, and Sb, were observed at relatively high concentrations (>1,000 μg/kg) in aerosols and aged e-liquids (Table S2) of the dPODs tested. Figure 3A and Table S8 present mean aerosol concentration data across all seven devices for Pb, Cr, Ni, Cu, Zn, and Sb from 100 to 500 puffs for ELF Bar and Flum Pebble devices and 100 to 300 puffs for Esco Bar devices due to Esco Bars losing function after 300 puffs. Aerosol concentrations of these elements between 100 and 500 puffs are presented in Figures S16–S22. In aged e-liquids of Esco Bar devices (Flavored and Clear), Pb, Ni, Cu, and Zn concentrations were excessively high ranging from 89,400 to 175,000 μg/kg Pb, 22,100 to 33,400 μg/kg Ni, 350,000 to 546,000 μg/kg Cu, and 308,000 to 462,000 μg/kg Zn (Figure S14, Table S2). In aerosols of Esco Bar devices (Flavored and Clear), Pb, Ni, Cu, and Zn concentrations were highest from 100 to 200 puffs (ranging from 3850 to 51,900 μg/kg Pb, 274 to 8930 μg/kg Ni, 4450 to 24,100 μg/kg Cu, and 13,100 to 87,500 μg/kg Zn; Table S3) and were significantly higher compared to the other devices tested for all puff ranges (100 to 1500). For comparison, in ELF Bar and Flum Pebble device aerosols from 100 to 200 puffs (see SI Section 2.4) concentrations of Pb, Ni, Cu, and Zn (≤35, ≤1520, ≤93, and ≤484 μg/kg, respectively; Table S3) were ∼1 to 3 orders of magnitude lower. The emission of Pb, Ni, Cu, and Zn in Esco Bar aerosols were rather uniform between 100 and 300 puffs (within 2-fold), with differences largely within the uncertainties of replicate measurements (Table S3, Figures S16 and S17). The comparatively high concentrations of these elements in Esco Bar device aerosols originate from nonheating components made of leaded bronze alloys (i.e., sheaths, wire connectors) (Figure 1), which were not observed in Flum Pebble and ELF Bar devices.

**Figure 3:**
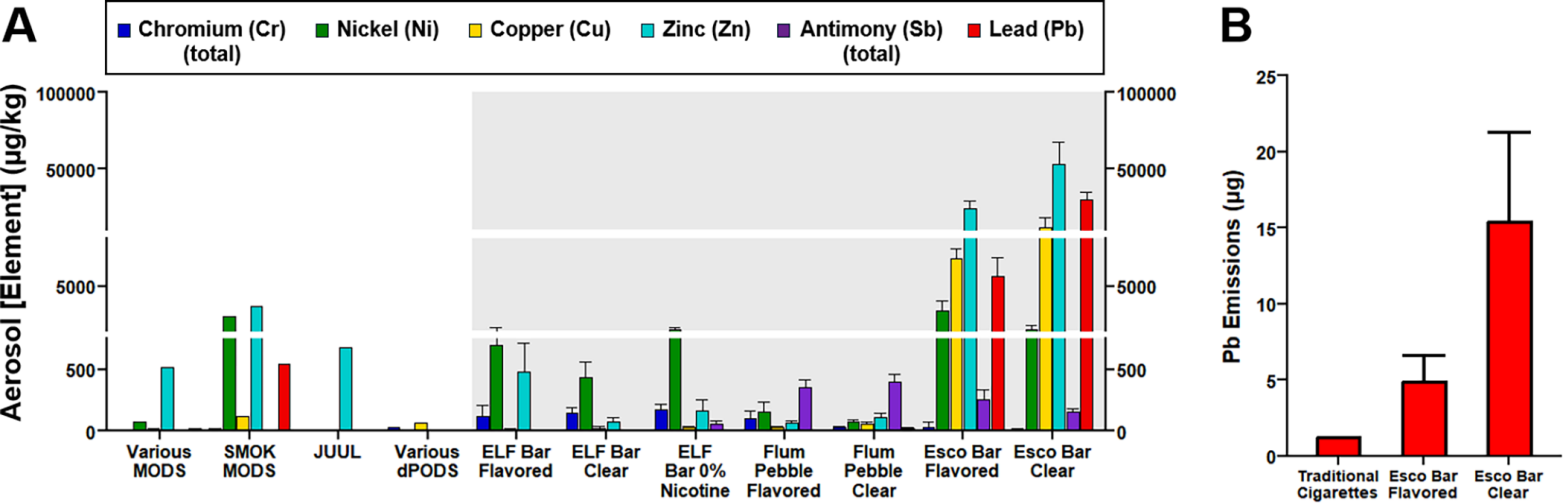
Comparisons between (A) aerosol element concentrations (total chromium (Cr), nickel (Ni), copper (Cu), total antimony (Sb), lead (Pb), and zinc (Zn)) between third and fourth generation e-cigarettes^7,18,41^ and disposable e-cigarettes (shaded region) tested in this study (mean aerosol concentrations from 100 to 500 puffs of each brand; ELF Bar, *n* = 9; Flum Pebble, *n* = 6; Esco Bar, *n* = 6) and (B) total Pb emissions between a pack of cigarettes^42^ and the equivalent nicotine dose of Esco Bar Flavored and Clear devices (this study). Third and fourth generation ENDS data included Olmedo et al. (third generation various MODs, *n* = 56),^18^ Zhao et al. (third generation SMOK MOD, *n* = 27; fourth generation JUUL, *n* = 9),^41^ and Aherrera et al. (fourth generation dPODs ZPOD, Bidi, and Stig, *n* = 23).^7^ See Table S9 for complete details. Error bars present standard deviations for triplicate aerosol collections (100–500 puffs).

Antimony (Sb), on the other hand, was present at high concentrations in aerosols from both Flum Pebble and Esco Bar brand devices (Figure 3A), with concentrations as large as 2300 μg/kg (Table S3). Concentrations of Sb in the aerosols of Flum Pebble and Esco Bar brand devices were modestly lower than virgin e-liquids at 100 puffs and increased linearly between 100 and 500 puffs (e.g., from 116 to 664 μg/kg Sb for the Flum Pebble Clear device; Table S3). All ELF Bar devices had universally low Sb concentrations in aerosols (≤10 μg/kg), aside from a single replicate of the ELF Bar Flavored 0% Nicotine product (94 to 271 μg/kg; Table S3). As the source of Sb was not identified, its elevated concentrations in virgin e-liquids are not predictable or constant with brand.

Arsenic (As), cadmium (Cd), tin (Sn), and bismuth (Bi) were detected at low concentrations in aerosols from ELF Bars and Flum Pebbles, with no trends across device ages (Table S3). This is consistent with their limited presence in device components (Figure 1, Table S1) and virgin e-liquids (Table S2). In contrast, Esco Bar e-liquids and aerosols contained elevated Cd, Sn, and Bi. Aerosol concentrations of Al, Fe, Mn, Se, Ba, and U were generally below the method limits of detection (LOD) and quantitation (LOQ) across all brands, thus aerosol data for these elements are not reported.

Metal and metalloid concentrations in disposable e-cigarette aerosols in this study were greater than those reported for second–fourth generation e-cigarette aerosols (Figure 3A, Table S9.1). A direct comparison across studies is often hindered by differences in how aerosol elemental concentration data are reported (e.g., as element mass/aerosol mass,^18,41^ element mass/puff,^8^ or element mass/air volume^7^); for this reason, we present disposable e-cigarette aerosol data in all relevant units (Tables S3 and S10–S12) and provide complete details on the comparisons between studies in SI Section S2.4. In brief, Pb, Cr, Ni, and Sb concentrations in all dPODs analyzed in this study exceeded those of the third generation MODs^18^ by up to 3 orders of magnitude (Table S9.2) and fourth generation JUUL aerosols^41^ by up to 4 orders of magnitude (Figure 3A, Table S9.2). Further, a direct comparison of the devices tested here to three other brands of dPODs (ZPOD, Bidi, Stig)^7^ shows that Cr, Ni, and Cu aerosol concentrations reported in this study were up to 2.2 orders of magnitude higher in some instances. Importantly, Pb, Sb, and Zn concentrations (μg/kg) were either not reported or below the limit of detection for some previously studied devices.^7^ The only published metal or metalloid concentrations data (Pb, Cr, Ni, Cu, Sb) comparable to those reported here are from Zhao et al.^41^ on a third generation MOD (brand SMOK) (Figure 3A), which documented element concentrations (Table S9.1) higher in some instances and lower in others instances than devices in this study. Notwithstanding, Pb concentrations in Esco Bar device aerosols were up to 55-fold greater than the third generation SMOK aerosols and Sb concentrations in both Flum Pebble and Esco Bar device aerosols were ≥83-fold greater than the third generation SMOK aerosols (Table S9.1).^41^ Comparisons of element concentrations between the dPODs in this study and those in previous research underscore the increased metal and metalloid exposure associated with prolonged use of dPODs, particularly in comparison to older generation e-cigarettes. The design of dPODs, which lack customization options such as e-liquid and coil exchanges, combined with metal accumulation over device life spans, is likely a significant factor contributing to the higher metal and metalloid emissions relative to earlier generations of e-cigarettes.^7,18,41^

To place the potential for Pb exposure in the context of traditional cigarettes, Figure 3B compares the mass of Pb in a pack of traditional cigarettes (20 cigarettes) to the mass of Pb of a comparable nicotine dose from the Esco Bar devices (details provided in SI Section S2.4). In comparison to the highest Pb delivery measured for traditional cigarettes, on average Esco Bar devices (Flavored and Clear) emitted ∼4 to 13 times more Pb (4.9 and 15.4 μg, respectively) in the first 200 puffs than the highest reported for a pack of cigarettes (20 cigarettes; 1.2 μg)^42^ (Figure 3B, Table S13.2). For context, this level of Pb exposure is equivalent to smoking as many as 19 packs of cigarettes in a single day (Table S13.3). Collectively, the findings in this work reveal that popular brands of disposable e-cigarettes (ELF Bars, Esco Bars, Flum Pebbles) among adolescent children^2,3^ emit more metals and metalloids in aerosols than older generations of e-cigarettes,^18,41^ other disposable e-cigarettes,^7^ and traditional cigarettes,^42^ originating from heating elements (that release Cr and Ni), leaded bronze alloys (that release Pb, Ni, Cu, and Zn), and unknown sources (of Sb), which warrants thorough cancer and noncancer risk assessments.

### Speciation of Chromium and Antimony in E-Cigarette Aerosols

Element speciation analyses were conducted on ELF Bar and Flum Pebble aerosols to quantitate the oxidation states of Cr (nontoxic Cr­(III) vs carcinogenic Cr­(VI))^40^ and Sb (carcinogenic Sb­(III) vs nontoxic Sb­(V))^38,43,44^ using liquid chromatography ICP-MS (Figure 4, Table S14), which is critical information for toxicological risk assessments. Hexavalent chromium, Cr­(VI), is a Group A carcinogen (U.S. EPA),^45^ while trivalent chromium, Cr­(III), is considered a nontoxic essential element.^46^ Trivalent antimony, Sb­(III), is classified as a possible carcinogen (Group 2A) by the International Agency for Research on Cancer (IARC)^44^ and is considered more toxic than pentavalent antimony Sb­(V).^43,44^ In freshly collected aerosols from Flum Pebble Flavored devices, Sb­(III) accounted for 33.5 ± 19.1% (ranging from 4.02 to 74.4% Sb­(III); Table S14) of the total Sb concentration on average across three replicates from 100 to 1200 puffs (example chromatogram shown in Figure 4B). In contrast, Flum Pebble Clear devices produced notably less Sb­(III), averaging 5.2 ± 3.5% (ranging from 0.0 to 13.1% Sb­(III); Table S14). Further, in freshly collected aerosols from ELF Bar and Flum Pebble devices (Flavored and Clear), Cr­(III) accounted for 100% of the total Cr (Figure 4A). Previous risk assessments of e-cigarette aerosols use total Cr concentrations in their calculations,^7,8,18^ which makes the assumption that all Cr in e-cigarette aerosols is carcinogenic Cr­(VI); however, that assumption is not supported by the data presented here of exclusive Cr­(III) in aerosols of dPODs and third and fourth generation devices.^47^ Moving forward, Cr and Sb speciation measurements are recommended for improved accuracy in risk assessments.

**Figure 4:**
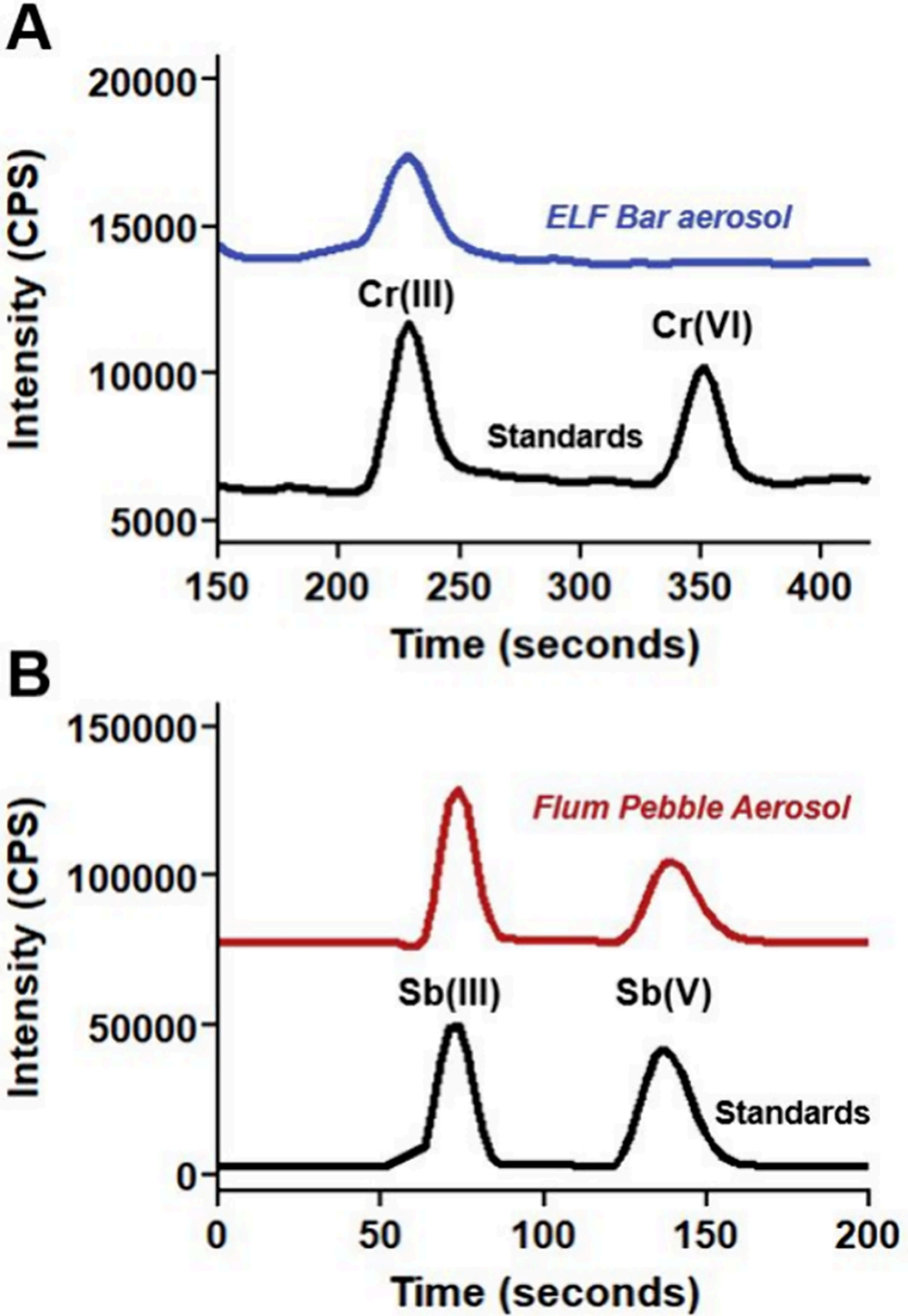
Chromatograms showing (A) chromium (Cr) distribution as solely Cr­(III) and (B) antimony (Sb) distribution as a mixture of 51% Sb­(III) and 49% Sb­(V) in e-cigarette aerosols. Reference standards are presented in each subplot.

### Disposable E-Cigarettes Pose Increased Noncancer and Cancer Risk to Daily Users

Cancer and noncancer risk assessments were conducted for all disposable e-cigarette devices using aerosol concentration data (between 100 and 500 and 100–1500 puffs) and oxidation-state information for the relative abundances of Sb and Cr species (complete details in the Methods Section and SI Sections S1.7 and S2.6, Figures S23–S24, and Tables S15–S16). Sb aerosol concentrations for Flum Pebble Flavored and Clear were adjusted by multiplying the total Sb concentrations by 33.5% and 5.2%, respectively, to account for the average relative abundances of Sb­(III) (Table S14). Cancer risk analyses of the two fully aged devices (100–1500 puffs; ELF Bar Flavored, ELF Bar Clear) and Esco Bar Flavored device (100 to 300 puffs) using averaged cancer risk values across all samples (Table S16) exceeded the cancer risk limit of 1 in 100,000 due to the extensive presence of respiratory carcinogen Ni (Figure 5A).^39,48,49^ Sb­(III) cancer risk assessments, after adjustments to the Flum Pebble Sb aerosol concentrations, yielded values exceeding the No Significant Risk Level (NSRL based on an acceptable risk limit of 1 in 100,000) of 0.13 μg/day^50^ up to 4-fold (Figure 5A, Table S16). Noncancer risk assessments show that mean Ni and Pb emissions of the three fully aged devices (ELF Bar Flavored, ELF Bar Clear, Flum Pebble Flavored) and Esco Bar devices (Clear and Flavored) between 100 to 300 puffs exceed the health quotient (HQ = 1) as high as 9-fold for Ni and 4-fold for Pb (Figure 5B, Table S16).^22,23,29^

**Figure 5:**
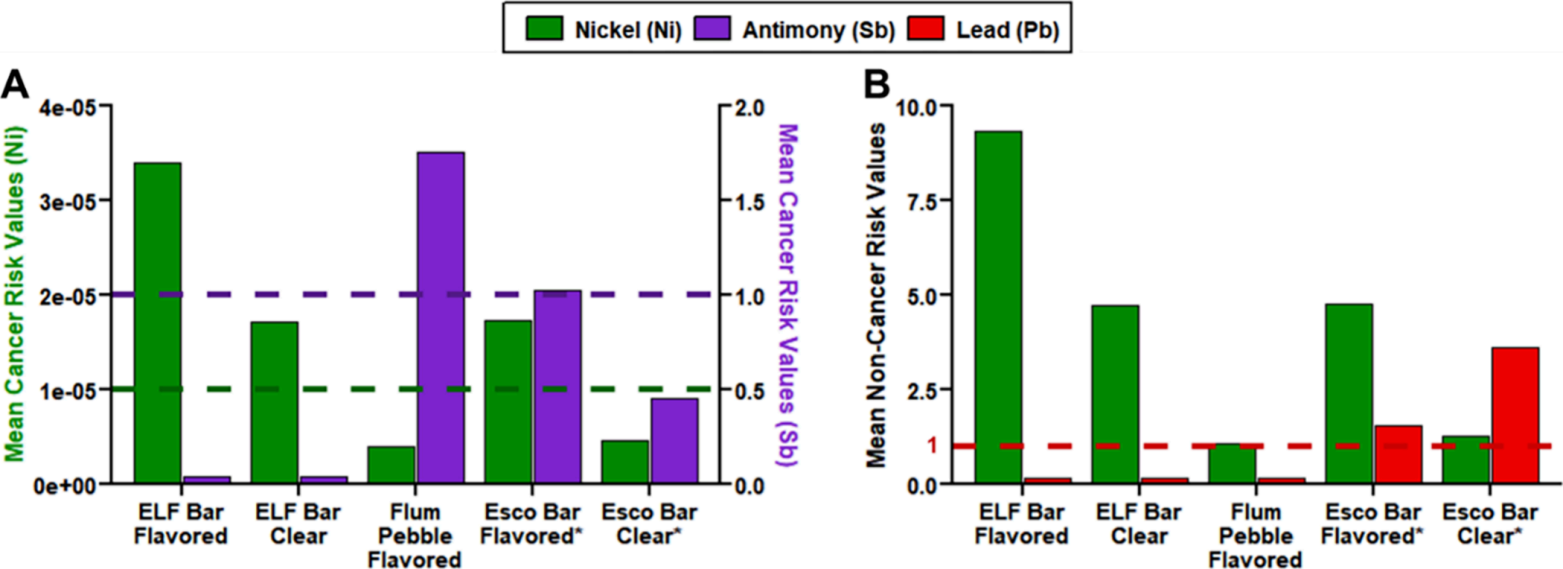
(A) Mean nickel (Ni) and antimony (Sb) cancer risk values and (B) Ni and lead (Pb) noncancer risk values of three fully aged devices (100–1500 puffs; ELF Bar Flavored, ELF Bar Clear, Flum Pebble Flavored) and Esco Bar Flavored device (100 to 300 puffs; Table S16, SI Section S1.7). Horizontal dashed lines in subplot A present cancer risk limits for Ni (green, 10^–5^) and Sb (violet, 1; based on CA OEHHA NSRL) and in subplot B present the noncancer risk limit (red; HQ = 1). Asterisks denote that Esco Bar values are derived from the 100 to 300 puff aerosol element concentrations.

This study combined three complementary ICP-MS methods (LA-ICP-MS, Q-ICP-MS, and LC-ICP-MS) to identify metal alloy compositions of internal components, measure total element concentrations in e-liquids and aerosols, and characterize redox-active metal and metalloid species present in the inhalable aerosols of popular disposable e-cigarette devices. We document concentrations of several toxic elements in disposable e-cigarettes that vastly exceed those in previous generations of e-cigarettes and traditional cigarettes (Figure 3),^7,18,41,42^ and Ni, Sb­(III), and Pb exceeded cancer and noncancer rick thresholds (Figure 5). To the best of our knowledge, this is the first study to determine Cr and Sb oxidation states in e-cigarette aerosols and e-liquids using LC-ICP-MS (Figure 4) and to incorporate element speciation into an e-cigarette aerosol risk assessment. Coupling the high element exposures and health risks associated with these devices and their prevalent use among the underage population,^2,3^ there is an urgent need for regulators to investigate this issue further and exercise regulatory enforcement accordingly. The inconsistencies found in the internal components of various dPOD brands, both within and between brands, highlight a significant lack of manufacturing and regulatory oversight. Specifically, the use of leaded bronze alloy components within Esco Bar brand devices, which leach Ni and Pb to e-liquids, poses both elevated noncancer and cancer risk based on daily use (Figure 5). Our study found one brand with hazardous material construction out of three popular brands tested. Yet there are nearly 100 unique disposable e-cigarette brands on the market currently,^51^ underscoring the need to better characterize the extent of the problem and implications for public health.

Further, Ni released from heating coils and Sb from contaminated e-liquids pose elevated cancer risks across the devices tested, as 33.5% of Sb was present as carcinogenic Sb­(III) in aerosols on average across three Flum Pebble Flavored devices (Figure 4B, Table S14). Nontoxic Cr­(III) was the only oxidation state of Cr observed in aerosols (Figure 4A), and thus Cr was omitted from risk assessment analyses. However, the potential oxidation of Cr­(III) to carcinogenic Cr­(VI) in the lungs should be evaluated due to possible toxicant exposure causing suppressed antioxidant capacity in the oxygen-rich environment of the lungs.^30,52,53^ Future studies are needed to evaluate metals and metalloid concentrations and emission profiles in aerosols across the rapidly evolving market of disposable e-cigarettes, identify the source(s) of Sb observed here in different brands of virgin e-liquids, assess how the composition of e-liquids (e.g., flavorings, acid type, nicotine content) and shelf life and storage conditions^6,54,55^ may impact metal and metalloid release and oxidation states, and quantify toxicity through *in vitro* and *in vivo* exposures.

## Methods

### Disposable POD (dPOD) E-Cigarette Devices

Three different brands of disposable e-cigarette devices (dPODs) were chosen based on their popularity: ELF Bar BC5000 (*n* = 9) (iMiracle Technology, Shenzhen, China), Flum Pebble 6000 (*n* = 6) (Flumgio Technology, La Puente, CA, USA), and Esco Bar 2500 (*n* = 6) (Pastel Cartel, Pflugerville, TX, USA) (Figure S1). All devices were ordered through online vendors in the United States. Flavored (Watermelon Ice, ELF Bar; Straw Mango, Flum Pebble; Tropical Rainbow Blast, Esco Bar) and “Clear” versions of each device were analyzed in triplicate. A 0% nicotine version of the ELF Bar BC5000 with the same flavoring (Watermelon Ice) was also analyzed to investigate the effect of nicotine on element emissions. All devices were kept at room temperature and were sampled within 2 weeks of purchase. E-liquids were extracted from the wicks of virgin, unused devices and analyzed for pH, density, and elemental content. See SI Sections S1.2 and S1.3 and Table S17 for more details.

### Laser Ablation ICP-MS of dPOD Components

All metallic components of dPOD devices in contact with e-liquids were analyzed for elemental composition by laser ablation ICP-MS (LA-ICP-MS) using an Agilent 8900 ICP-MS instrument (Agilent Technologies, Palo Alto, CA) in single quadrupole mode coupled to a New Wave UP213 laser (New Wave Research, 48660 Kato Road, Freemont CA 94538), including wire coils, mesh coils, sheaths, wire coil coatings, mesh coil supports, battery connectors, and wiring solder (Figures S1–S9). Components in contact with the e-liquids were removed and rinsed in ultrahigh purity water (≥18.2 MΩ cm) followed by methanol prior to analysis. The relative abundance of elements was determined in time-resolved analysis (TRA) mode. See SI Section S1.1 and Tables S1 and S18 for complete details.

### Aerosol Generation and Collection

Weights were recorded of the devices before (MD_B_ = mass of device precollection) and after aerosolization (MD_A_ = mass of device postcollection) to measure the mass of aerosol generated by the device (eq 1).

1 $$total\;aerosol\;generated\;\left(g\right)={MD}_{B}-{MD}_{A}$$

Devices were puffed twice a minute for 2 s per puff at a flow rate of 1.70 mL/min, which is equivalent to a puff volume of 56.7 mL (eq 2).

2 $$puff\;volume\;\left(mL\right)=\frac{1.7\;L}{\min}\times \frac{1\;\min}{60\;\sec}\times \frac{2\;s}{puff}\times \frac{1000\;mL}{1\;L}$$

Aerosol samples were collected using 6 mL syringes filled with 0.35 g of raw quartz wool fibers (9–30 μm Coarse Quartz Wool, Thermo Scientific, Fair Lawn, NJ, USA) connected to a vacuum line and modulated by solenoid valves with a time relay controller (PTR4-SP, Changzhou Xuchuang Info. Tech. Co., Changzhou, China). The puff volume of 56.7 mL was within the range of puff volumes reported for other e-cigarettes^56^ and was chosen based on preliminary experiments which observed that oversaturation of the quartz wool occurred at higher puff volumes leading to loss of aerosol collection efficiency (SI Section S1.3). Devices were fully charged before each use. See SI Section S1.3 for additional details.

All dPOD brands and flavors were puffed in 100 puff intervals to 500 puffs to conduct aging analyses of the first 500 puffs (*n* = 3 per device type). Aged e-liquid samples were collected from devices after 500 puffs to assess changes in element concentrations in e-liquids with device use. A single device of ELF Bar Flavored, ELF Bar Clear, and Flum Pebble Flavored were chosen for a full aging analysis (*n* = 1 per device type) from 100 to 1500 puffs, which measured element emissions across the full life span of the devices.

### Aerosol and E-Liquid Multi-Element Analysis

Microwave digestion was conducted on e-liquid, aerosol samples, and quartz wool blanks using a Milestone ETHOS UP microwave digester (Milestone Srl, Fatebenefratelli, Italy) with dilute HNO_3_ (60–70% Omni Trace, Merck Millipore, Darmstadt, Germany) and H_2_O_2_ (30% Trace Metal Grade, Sigma-Aldrich, St. Louis, MO, USA) solutions to oxidize organic carbon in samples. See SI Section S1.4 for a thorough process description. The use of microwave digestion was proven to be effective during method development at reducing carbon-based polyatomic interference and stabilizing internal standard recoveries as compared to undigested aerosol samples. Multielement analysis was conducted using a Thermo-Fisher iCAP RQ ICP-MS (Thermo Fisher Scientific, Waltham, MA, USA) with a CETAC Teledyne ASX-560 autosampler (Teledyne Technologies, Rancho Cordova, CA USA) for the following elements: aluminum (Al), As, Ba, bismuth (Bi), Cd, Cr, Cu, Fe, Mn, Ni, Pb, Sb, Se, Sn, U, and Zn (see SI Section S1.5 for limits of detection (LOD) and quantitation (LOQ)). Calculations for aerosol and e-liquid element concentrations, masses, mass per 100 puffs, and air concentrations per 100 puffs are presented in SI Section S1.4. Complete details of statistical analyses are provided in SI Section S1.8.

### Chromium (Cr) and Antimony (Sb) Speciation Analysis

Liquid chromatography ICP-MS was conducted on diluted aerosol samples using a Thermo Fisher iCAP RQ ICP-MS (Thermo Fisher Scientific, Waltham, MA, USA) coupled with an Agilent 1100 binary pump (G1312A), autosampler (G1313A) (Agilent Technologies, Santa Clara, CA, USA), PRP-X100 Anion Exchange HPLC Column (4.1 mm × 50 mm, 5 μm; Hamilton Company, Reno, NV, USA), and PRP-X100 Guard Cartridge, PEEK (Hamilton Company, Reno, NV, USA) to quantify the distribution of Cr­(III) versus Cr­(VI) and Sb­(III) versus Sb­(V). Method validations were conducted using calibration ranges of 0.1–10 μg/L for Sb­(III) and Sb­(V) and 0.1–10 μg/L for Cr­(III) and Cr­(VI) with 1 μg/L spiked e-liquid (2% nicotine, 1:1 benzoic acid in 50:50 PG:VG). Working standards at 1 μg/L of each Sb and Cr species were used to assess recovery and were within 80–120%. See SI Section S1.6 for complete method details on mobile phase, flow rates, and sample preparations (Table S18).

### Cancer and Noncancer Risk Assessment Analyses

Noncancer and cancer risk assessments were conducted to estimate metal exposure health risks from dPOD usage over the life span of each device using mean air concentrations (mg/m^3^) for every 100 puffs from 100 to 500 puffs for each dPOD analyzed and for every 100 puffs of each device in the full aging analysis. Complete details are provided in SI Section 1.7. For relevance to the nicotine consumption of a pack of cigarettes per day, 100 puffs per day was used as the estimated user puff profile exposure parameter for risk assessment (Tables S15 and S16), which is similar to reported average use of e-cigarette users (115–140 puffs per day).^7,57,58^ 100% absorption was assumed for elements measured in the aerosols. Cr, Ni, As, Cd, Sb, and Pb were the only elements included in the risk assessment due to their potential carcinogenic properties (Table S16).^28,46,49^ Based on the speciation results (Table S14), Sb concentrations were multiplied by the average relative abundance of Sb­(III) for Flum Pebble Flavored and Clear aerosols (33.5% and 5.2%, respectively) to accurately assess the risks associated with Sb­(III) exposure. Noncancer health risk is reported as health quotients (HQ) in Table S16.

## Supplementary Material





## Notes

Additional experimental details, calculations, materials, and methods, including tabulated data and element e-liquid and aerosol concentrations, photographs of devices and laser ablation samples, and supplementary data plots for aerosol mass generation, and cancer risk evaluations (PDF)
Tables S1–S22 (XLSX)
