# Supplementary material for: Elevated Toxic Element Emissions from Popular Disposable E‑Cigarettes: Sources, Life Cycle, and Health Risks
Source: ACS Cent Sci. 2025 Jun 25;11(8):1345–54. doi: 10.1021/acscentsci.5c00641 (PMC12395296; doi:10.1021/acscentsci.5c00641)
Supplement: Supplementary file 1 [file oc5c00641_si_001.pdf]

Supporting Information for

Elevated toxic element emissions from popular disposable e-cigarettes: sources, life cycle, and health risks

Mark R. Salazar[1], Lalima Saini[1], Tran B. Nguyen[1], Kent E. Pinkerton[2], Amy K. Madl[2], Austin M. Cole[3], Brett A. Poulin[1*]

[1]Department of Environmental Toxicology, University of California Davis, Davis, CA 95616, United States

[2]Center for Health and the Environment, University of California Davis, Davis, CA 95616, United States

[3]Interdisciplinary Center for Plasma Mass Spectrometry, University of California Davis, Davis, CA 95616, United States

* Corresponding author. Tel: +1 530 754 2454. Email address: bapoulin@ucdavis.edu

Supporting information includes 24 Figures, 22 Tables and 45 pages. See **SI Tables** spreadsheet for supplementary tables (Tables S1-S22).

ORGANIZATIONAL AFFILIATIONS RELEVANT TO FINANCIAL COMPETING INTERESTS

One of the authors (AKM), in addition to an appointment at the University of California, Davis, is employed by a scientific consulting firm, Valeo Sciences LLC, which provides scientific advice to the government, corporations, law firms, and various scientific/professional organizations. AKM has been engaged by various electronic nicotine delivery system (ENDS) and e-liquid manufacturers to provide general consulting and expert advice on scientific matters in litigation and in the context of regulatory requirements. All other authors declare no conflicts of interests. This research was not funded by any private corporations. This article was prepared and written exclusively by the authors without review or comment by any outside organization.

Table of Contents

Section 1: Supplemental Methods 3
S1.1 Laser Ablation Coupled ICP-MS (LA-ICP-MS) 3
S1.2 Disposable POD E-Cigarette (dPOD) E-Liquid Properties and Nicotine Contents 4
S1.3 Aerosol Collection 4
S1.4 Microwave Digestion 5
S1.5 Single Quadrupole Inductively Coupled Plasma Mass Spectrometry (Q-ICP-MS) 7
S1.6 Liquid Chromatography Coupled ICP-MS (LC-ICP-MS) 10
S1.7. Cancer and Non-Cancer Risk Assessment Analyses 14
Section 2: Supplementary Results & Discussion 16
S2.1 Virgin E-Liquid Analysis 16
S2.2 Full Aging Analyses (to 1500 puffs) 17
S2.3 Aged e-liquid & 500-puff Aerosol Analysis 18
S2.4 Aerosol Element Emission Comparisons 19
S2.5 LC-ICP-MS Cr & Sb Speciation 21
S2.6 Cancer and Non-Cancer Risk Assessment Analyses 22
Section 3: Supplementary Figures 25
SI References 44

Section 1: Supporting Methods

S1.1 Laser Ablation Coupled ICP-MS (LA-ICP-MS)

Coils, sheaths, battery connectors, and e-cigarette computer chips were analyzed by the Interdisciplinary Center for Plasma Mass Spectrometry at the University of California, Davis using an Agilent 8900 ICP-MS (Agilent Technologies, Palo Alto, CA) in single quadrupole mode coupled to a New Wave UP213 laser (New Wave Research, 48660 Kato Road, Freemont CA 94538). See **Table S18** for materials and instrument analysis parameters. Element abundances were quantified using LA-ICP-MS in time-resolved analysis (TRA) mode. The ICP-MS was operated at 1550 W forward power and the frequency quintupled Nd:YAG laser, wavelength = 213 nm, was operated at 0.227 mJ to ablate 40 µm spots with 18.07 J/cm^2 fluence at a pulse rate of 10 Hz. He gas was used to transport ablated material to the ICP-MS at 0.9 L/min, where Ar was added as a makeup gas at 1.2 L/min. Samples were analyzed in He mode, with He used as the collision/reaction cell (CRC) gas at 2.8 mL/min. Samples and quality control (QC) standard NIST 610 (National Institute of Standards and Technology, 100 Bureau Drive, Stop 2300, Gaithersburg, MD 20899) were mounted in 2.5 cm epoxy rounds sand analyzed in triplicate. NIST 610 was analyzed initially as QC to verify the calibration. All samples were analyzed in triplicate (*n* =3). See **Table S1** for a comprehensive summary of all element compositions. Background He/Ar gas signal was subtracted from acquired data. Single element intensities in counts per second (CPS) were divided by the sum of all element intensities measured and multiplied by 100% to determine the percentage of all elements in each sample (**Equation S1**). Standard deviations of single element measurements (CPS) were divided by the sum of all element intensities measured and multiplied by 100% to determine the percentage of standard deviation of each measurement (**Equation S2**). No calibration standards were used for elemental quantification, thus the data presented is qualitative.

$$\%\ of\ Elemental\ Composition = \frac{Single\ Element\ Intensity\ (CPS)}{\Sigma\ [All\ Element\ Intensities\ (CPS)]} \times 100\% \qquad \text{(S1)}$$

$$\%\ STDEV = \frac{Single\ Element\ Intensity\ STDEV\ (CPS)}{\Sigma\ [All\ Element\ Intensities\ (CPS)]} \times 100\% \qquad \text{(S2)}$$

S1.2 Disposable POD E-Cigarette (dPOD) E-Liquid Properties and Nicotine Contents

Virgin e-liquids from all devices were analyzed for pH and density (g/mL) (**Table S17**). E-liquid pH was measured using a FiveEasy Plus pH meter FP20 (Mettler-Toledo AG, Analytical, Greifensee, Switzerland), calibrated before use. E-liquid densities were measured by weighing 0.5 mL of e-liquid on an analytical balance and dividing the mass by volume. Virgin e-liquid pH values ranged from 4.36 – 4.68, indicating acidic e-liquids. e-Liquid densities for all devices ranged from 1.11 – 1.14 g/mL and were used to calculate the nicotine mass per device (**Table S17**). The generation of aerosol mass per 100 puffs, from 100-500 puffs, was calculated for each device using the total aerosol mass generated by the device divided by 100 puffs for each collection (n = 3) (**Table S17**). Flum Pebble devices generated the most aerosol mass per puff while Esco Bar devices produced the least and varied the most between replicate devices. The variability of Esco Bar aerosol mass production can be explained by the loss of power over the span of 500 puffs with the batteries of these devices often dying before reaching 500 puffs.

Flum Pebble ENDS contained the largest mass of nicotine per device at 700 mg, followed by ELF Bar ENDS at 380 – 475 mg, and Esco Bar ENDS at 300 mg (**Table S17**). When comparing the total nicotine contents (mg) to combustion cigarettes (1 pack contains 20 cigarettes on average with 1.5 mg of nicotine absorbed by the user per cigarette)[1] and assuming 100% nicotine absorption, Flum Pebbles contain 23 pack equivalents of nicotine, ELF Bar devices contain 13 – 16 pack equivalents of nicotine, and Esco Bar devices contain 10 pack equivalents of nicotine (**Table S17**). The average puff count across all seven ENDS was calculated by determining the number of puffs needed to emit 1.5 mg of nicotine and multiplying by 20 (cigarettes per pack). The average puff count equivalent of 1 pack of cigarettes for these ENDS ranged from 78-164 puffs (average ± standard deviation of 106 ± 32 puffs; **Table S17**). For simplification of the risk assessment conducted in this study, 100 puffs were used as the pack of cigarettes per day puff equivalent.

S1.3 Aerosol Collection

The mass of quartz wool per syringe filter was determined by comparing aerosol mass recovery between a range of 0.1 – 0.5 g of quartz wool at a flow rate of 1.7 L/min. It was determined that 0.35 g of quartz wool collected approximately 0.25 g of aerosol per 100 puffs without decreasing the flow rate. This

aerosol mass target range was selected to ensure that elemental concentrations could be quantified after sample digestion and dilution (dilution factor = 80). A consideration for this target mass is the generation of carbon dioxide gas ($CO_{2(g)}$) during microwave digestion of organic material. Organic masses > 0.5 g may be hazardous due to the reactivity of concentrated nitric acid and hydrogen peroxide with organic material producing unsafe gas pressure within digestion vessels. 0.25 g aerosol mass was appropriate to provide sufficient analyte signal and be safe to digest by microwave digestion. Some devices in this study produced higher than average aerosol mass per puff leading to aerosol mass collected with the quartz wool surpassing 0.25 g. We observed that our microwave digestion method was capable of safely digesting larger aerosol masses.

Aerosol collection efficiency was calculated as the ratio of aerosol mass collected to total aerosol generated multiplied by 100%. The mean aerosol efficiency across all collections in this study was 47%, lower than recoveries reported in previous ENDS studies using other collection methods such as solvent-free condensate.[2–4] Experiments using 4th generation POD-MOD e-cigarettes were conducted in which aerosol accumulated in the tubing past the quartz filter was collected (termed the “post-filter residual aerosol”), analyzed for element contents and compared to the concentrations in the quartz filter-collected aerosols. Chromium (Cr), iron (Fe), nickel (Ni), and zinc (Zn) residual aerosol concentrations, the only elements averaging greater than 20 µg/kg (ppb) in aerosols, were measured at 10.8 ± 14.2, 5.64 ± 6.05, 10.2 ± 15.3, and 20.9 ± 30.6% recovery, respectively, of the concentrations from aerosol collected by the syringe quartz wool filter (**Table S19**). This finding supports that quartz wool is effective at scrubbing aerosols of cations as aerosols pass through, depositing and concentrating elements on the quartz wool.

S1.4 Microwave Digestion

Due to the presence of organic constituents in the e-liquid and aerosol samples (i.e. nicotine, organic acids, flavorings), microwave digestion was used to decrease carbon-based polyatomic interference[5] by oxidizing carbon to $CO_{2(g)}$ with heated, concentrated nitric acid (HNO_3) and hydrogen peroxide (H_2O_2), thus effectively removing carbon from the samples. To prepare aerosol samples for digestion, aerosol-saturated quartz wool filters were carefully removed from the syringe using a plastic spatula and transferred to 100 mL MAXI-44 PTFE digestion vessels (Milestone Srl, Fatebenefratelli, Italy). E-liquid

samples were prepared by transferring and weighing 0.250 g of e-liquid in a digestion vessel. A 5 mL solution of 20% HNO_3 (v/v) (60-70% Omni Trace, Merck Millipore, Darmstadt, Germany) and 20% H_2O_2 (v/v) (30% Trace Metals Grade, Sigma Aldrich, St. Louis, MO, USA) in ultra-high purity water (>18.2 Ω cm; GenPure Pro UV Barnstead Milli-Q, Langenselbold, Germany) was added to each sample. Ultra-pure water was the first solvent added, followed by HNO_3 and then H_2O_2. Concentrated H_2O_2 can react violently with organic molecules, and therefore adding H_2O_2 last was the safest approach. Aerosol and e-liquid samples were digested at 200° C for 30 minutes and allowed to cool to room temperature (**Table S18**). Digested samples were transferred to 50 mL Falcon tubes (Corning Science, Tamaulipas, Mexico) using an Eppendorf automatic pipette (Xplorer 50-1000 µL, Hamburg, Germany) to record the volume transferred. Using the automatic pipette, ultrapure water was added to the solution to bring the solution to a final volume of 20 mL. Finally, aerosol sample solutions extracted from quartz wool were syringe filtered using 10 mL disposable syringes (Exel International, Redondo Beach, CA, USA) coupled with a 0.45 µm syringe filter (Whatman GD/XP 25, Cytiva, Buckinghamshire, UK) to remove quartz fibers to prevent nebulizer clogging and sample matrix interference. E-liquid samples were not subject to syringe filtration. Method blanks for aerosol samples and e-liquids were prepared in the same manner as described above with and without virgin quartz wool, respectively. One method blank was prepared per each set of microwave digestions.

Calculations for aerosol and e-liquid element concentrations, masses, mass per 100 puffs, and air concentrations per 100 puffs are shown below where M_A and M_E are the aerosol or e-liquid element concentration (µg/L), respectively, M_{Blank} is the method blank element concentration (µg/L), V_F is the final volume (0.02 L) of the sample solution, T_A is the mass of total aerosol generated by the e-cigarette (g), and 20 m^3 is the general daily inhalation rate provided by the US Environmental Protection Agency's (U.S. EPA) Exposure Factors Handbook.[6] Element concentrations below the limit of quantification for method blanks were excluded from blank subtraction calculations.

$$Element\,Concentration\ (\mu g/kg) = \left(M_{A/E} - M_{Blank}\right) \times V_F \times \frac{1}{T_A} \times \frac{1000\ g}{1\ kg} \qquad (S3)$$

$$Element\,Mass\ (ng) = \left(M_A - M_{Blank}\right) \times V_F \times \frac{1000\ ng}{1\ \mu g} \qquad (S4)$$

$$Element\ Mass\ per\ Puff\ (ng/puff) = (M_A - M_{Blank}) \times V_F \times \frac{1000\ ng}{1\ \mu g} \times \frac{1}{100\ puffs} \qquad (S5)$$

$$Element\ Air\ Concentration\ (mg/m^3) = ng\ of\ element\ (S4) \times \frac{1}{20\ m^3} \times \frac{1\ mg}{1\ x\ 10^6 ng} \qquad (S6)$$

Hydrogen peroxide (H_2O_2) was used in this solution composition to enhance oxidation of the aerosol organic carbon and maintain a low dilution factor. Preliminary digestion method development tests showed that 20% HNO_3 solutions alone were ineffective at reducing internal standard interference. A scenario of digesting at higher HNO_3 concentration would result in a larger dilution factor needed to maintain a final solution composition of 5% HNO_3, which was not ideal given low concentrations of metals reported in some e-cigarette aerosols.[2,3,7] In addition, the use of higher concentrations of HNO_3 in the sample matrix for ICP-MS analysis are not ideal due to polyatomic interferences from nitrogen-based compounds.[5] By using concentrated H_2O_2 in the digestion solution as a substitute for an additional 20% HNO_3, the oxidation power of the digestion solution was increased while the dilution factor was maintained at 80. It is worth noting that the oxidation power of this digestion solution is not maximal since the solution is dilute, and likely some of the organic carbon was likely not oxidized completely to $CO_{2(g)}$. However, the digestion conditions (20% HNO_3 and 20% H_2O_2) solution oxidized enough of the organic carbon in the sample matrix to effectively analyze sample solutions without internal standard recovery issues.

S1.5 Single Quadrupole Inductively Coupled Plasma Mass Spectrometry (Q-ICP-MS)

Multi-element analysis was conducted using a Thermo-Fisher iCAP RQ ICP-MS (Thermo Fisher Scientific, Waltham, MA, USA) with a CETAC Teledyne ASX-560 autosampler (Teledyne Technologies, Rancho Cordova, CA USA) for aluminum (Al), arsenic (As), barium (Ba), bismuth (Bi), cadmium (Cd), chromium (Cr), copper (Cu), iron (Fe), manganese (Mn), nickel (Ni), lead (Pb), antimony (Sb), Se, tin (Sn), uranium (U), and zinc (Zn). See **Table S18** for materials and instrument analysis parameters. Ultra-high purity (99.99%) argon gas was used as the plasma and carrier gas at a flow rate of 1 L/min. Helium was used as the collision cell gas at a flow rate of 4.5 mL/min and kinetic energy discrimination (KED) mode was used for all isotopes. The instrument was tuned before each analysis with cerium (Ce) oxide

ratios < 1.5% ($^{156}CeO^+$ / 140Ce$^+$) and < 3.0% doubly charged ions ($^{70}Ce^+$ / $^{140}Ce^{++}$) to ensure optimal sensitivity and signal stability.

Two custom-made ICP-MS combination standards containing Al, As, Cd, Cr, Cu, Fe, Mn, Ni, Pb, Sb, Sn, U, and Zn at 10 µg/mL were used for calibration and independent quality control (QC) standards (Inorganic Ventures, Christiansburg, VA, USA; AccuStandard, New Haven, CT, USA). Single-element ICP-MS standards for Ba, Bi, and Se at 10 µg/mL were added to the calibration and independent QC standard solutions (Inorganic Ventures, Christiansburg, VA; High Purity Standards, North Charleston, SC). Single-element ICP-MS standards for scandium (Sc), yttrium (Y), and terbium (Tb) at 10 µg/mL were used for the internal standard solution (Inorganic Ventures, Christiansburg, VA, USA) and added to sample injections at 1 µg/L using a peristaltic pump and T-junction mixer. All standards were prepared with 5% HNO_3 (v/v), 5% H_2O_2 (v/v) in Milli-Q ultrapure water solution. H_2O_2 could have been omitted from the diluent since H_2O_2 degrades to H_2O extensively at high temperatures, which likely occurred during sample microwave digestion. However, internal standard recoveries were stable for all samples and standards; therefore, the addition of H_2O_2 in the standard solutions did not impact data quality. Two QC standards were injected at 1 and 10 µg/L to verify accuracy of results. All samples were injected in brackets of 10 samples followed by a carryover assessment blank, two independent QC standards prepared at 1 and 10 µg/L, and a second carryover assessment blank. Carryover blank element concentrations were all less than the concentration of the lowest calibration standard. All independent QC standard elements recovered within 80 – 120% suggesting accurate results. Internal standard recoveries were within 80-120% for all samples and standards, indicating the negligible impact of matrix effects and instrument drift.

Calibration ranges of 0.001 – 100 µg/L for Cd, Sb, Pb, Bi, and U, 0.01 – 100 µg/L for As, Se, Sn, and Ba, 0.1 – 100 µg/L for Cr, Mn, Ni, and Cu, and 1 – 100 µg/L for Al, Fe, and Zn. Ranges varied per element due to higher background noise for some elements at the lower end of the calibration curve. The coefficient of determination (R^2) was greater than 0.995 for each calibration curve. Instrument limits of determination (LOD) and limits of quantitation (LOQ) determined across all 13 analyses using QTEGRA software (Thermo Fisher Scientific, Waltham, MA, USA) and were reported across all 16 analytes (**Table**

S20). Instrument LOD was calculated by multiplying the standard deviation of the calibration blank by 3 and dividing by the slope of the calibration function. Instrument LOQ was calculated by multiplying the standard deviation of the calibration blank by 10, dividing by the slope of the calibration function, and multiplying by the total dilution factor.

To quantify the contributions of metals from the laboratory air, air blanks were collected in the same manner as aerosol samples by drawing 100 puffs through quartz wool syringes without attached devices and microwave digested as described in Section S1.4. (*n* = 6; Table S21). A single quartz wool blank, not puffed but prepared in the same manner as the air blanks, was also prepared and analyzed alongside the six air blanks (*n* = 1). Method blank (*n* = 15) and air blank concentrations (in µg/kg) were combined for statistical analysis, and all relevant data are presented in **Table S21**. Method detection limits (LOD) and quantitation limits (LOQ) for each element were determined by multiplying the standard deviation of blank measurements, collected across multiple days, by factors of 3 and 10, respectively. Method LODs (µg/kg) were 322.9 (Al), 25.2 (Cr), 10.8 (Mn), 386.1 (Fe), 12.5 (Ni), 26.0 (Cu), 97.3 (Zn), 0.4 (As), 9.3 (Se), 0.2 (Cd), 66.1 (Sn), 1.4 (Sb), 144.8 (Ba), 0.5 (Bi), 0.0 (U), 17.8 (Pb) and method LOQs (µg/kg) were 1076.3 (Al), 84.1 (Cr), 35.9 (Mn), 1286.8 (Fe), 41.7 (Ni), 86.6 (Cu), 324.4 (Zn), 1.5 (As), 31.1 (Se), 0.5 (Cd), 220.2 (Sn), 4.5 (Sb), 482.6 (Ba), 1.8 (Bi), 0.2 (U), 59.2 (Pb) (**Table S21**). The majority of aerosol concentrations of Al, Fe, Mn, Se, Ba, and U were either below method limits of detection (LOD) and quantitation (LOQ) across all brands and were thus not reported for aerosols. Elements of toxicological interest (Cr, Ni, Cu, Zn, As, Cd, Sn, Bi, and Pb) exceeding the method limit of quantitation (LOQ) were reported; however, in some cases, values below the LOQ or even the limit of detection (LOD) were also included due to their toxicological relevance and notably high concentrations in specific brands. These values are flagged as estimates in **Tables S3, S8,** and **S10–S12**.

A method validation was performed using triplicate spiked samples, which included adding spiked e-liquid samples (50 µg/L per each element) to 0.35 g of quartz wool (**Tables S22.1–S22.3**). Spiked samples were processed using the microwave digestion method described above. 2% nicotine, 1:1 benzoic acid in 30:70 PG:VG e-liquid was used in place of aerosol to evaluate the spike recovery using a sample of similar composition to aerosols. Mean spike recoveries for all 16 elements ranged from 90.4 –

111.9% with a maximum standard deviation of 5.5%, validating suitable metal recovery with this method (**Table S22.3**).

S1.6 Liquid Chromatography Coupled ICP-MS (LC-ICP-MS)

Cr and Sb speciation methods were adapted from previously published LC-ICP-MS methods for quantitating Cr and Sb species in natural waters.[8,9] Speciation analyses were performed using a Thermo Fisher iCAP RQ ICP-MS (Thermo Fisher Scientific, Waltham, MA, USA) coupled with an Agilent 1100 binary pump (G1312A) and autosampler (G1313A) (Agilent Technologies, Santa Clara, CA, USA). Two distinct methods, tailored to each set of species, were applied using the same instrumentation but with different parameters. PEEK capillaries, needle seat, and rotor seal were used for corrosion resistance. See **Table S18** for materials and instrument analysis parameters. A Burgener PEEK Mira Mist Nebulizer (Burgener Research, Ontario, Canada) was utilized to manage the HPLC flow rate of 1.2 mL/min employed in each speciation method. Ultra-high purity (99.99%) argon gas was used as plasma and carrier gas at a flow rate of 1 L/min. The ICP-MS was tuned before each analysis with cerium (Ce) oxide ratios < 1.5% ($^{156}CeO^+$ / 140Ce^+) and < 3.0% doubly charged ions ($^{70}Ce^+$ / $^{140}Ce^{++}$) to ensure optimal sensitivity and signal stability. Helium was employed as the collision cell gas in kinetic energy discrimination (KED) mode for Cr speciation analyses, with the flow rate manually optimized to 2 mL/min to maximize the Cr signal-to-noise ratio. For Sb speciation analysis, standard (STD) mode was used, as optimization tests demonstrated that STD mode delivered superior apex peak height-to-baseline values and peak resolution compared to any KED mode flow rate tested.

A PRP-X100 Anion Exchange HPLC Column (4.1 x 50 mm, 5 µm; Hamilton Company, Reno, NV, USA) and PRP-X100 Guard Cartridge, PEEK (Hamilton Company, Reno, NV, USA) were used for separation of Cr and Sb Species. An HPLC flow rate of 1.2 mL/min was identified as optimal for both methods. Both methods utilized an isocratic mobile phase gradient. Sample injection volumes of 20 µL for Sb and 40 µL for Cr speciation provided the best peak-to-baseline ratios and peak resolution for their respective species. Both methods incorporated a 100 µL PEEK sample loop. Isotopes Sc^{45}, Cr^{52}, Y^{89}, and Sb^{121} were analyzed for each method, with data acquisition intervals set at 0.2 seconds per point. Run

times of 3.33 (200 seconds) and 7 minutes (420 seconds) were used for Sb and Cr methods, respectively. All samples were aliquoted to 2 mL polypropylene HPLC vials (Agilent Technologies, Santa Clara, CA, USA) for injection. Sb samples were diluted 2-fold in the HPLC vials by adding 0.5 mL of sample solution to 0.5 mL of 5 mM citric acid and mixing before injection. Preliminary experiments for Cr speciation identified that repeat injections into the same vial caused carryover and adversely impacted the quality of measurements. All polypropylene HPLC vials were soaked overnight in 5% HNO3 and fully dried to reduce background contamination.

Cr speciation mobile phase and sample diluent (0.5 mM EDTA in ultrapure water, pH 7.0) was prepared by adding 0.404 g of trace metals grade ethylenediaminetetraacetic acid dipotassium salt dihydrate (EDTA, Sigma-Aldrich, St. Louis, MO, USA) to 2 L of ultra-pure water and pH adjusted to 7.0 using NH_4OH (10% ammonium hydroxide, Thermo Scientific, Ward Hill, MA, USA) and concentrated HNO_3. 500 mL of mobile phase solution was transferred to a separate container and spiked with Sc ICP-MS standard at 1 μg/L to produce sample diluent solution. Cr standards ranging from 0.1 μg/L to 10 μg/L were prepared using serial dilutions of 1000 μg/mL Cr(III) and Cr(VI) ICP-MS standards (Inorganic Ventures, Christiansburg, VA, USA) with sample diluent solution.

Sb speciation mobile phase (10 mM EDTA, 1 mM Phthalic Acid in ultrapure water, pH 4.5) was prepared by adding 8.09 g of ethylenediaminetetraacetic acid dipotassium salt dihydrate (EDTA) and 0.332 g of phthalic acid (Sigma-Aldrich, St. Louis, MO, USA) to 2 L of ultra-pure water and pH adjusted to 4.5 using 10% NH_4OH and concentrated HNO_3. 20 μL of 10 μg/mL Y ICP-MS standard added to the solution to monitor baseline fluctuations. Sb sample diluent solution (5 mM Citric Acid in ultra-pure water) was prepared by adding 1.92 g of citric acid (Sigma-Aldrich, St. Louis, MO, USA) to 2 L of ultra-pure water. A Sb(III) stock standard at 1000 μg/mL was prepared by adding 0.134 g of potassium antimony(III) tartrate hydrate (Sigma-Aldrich, St. Louis, MO, USA) to 50 mL of 5 mM citric acid. A Sb(V) stock standard at 10 μg/mL was prepared by adding 0.0202 g of potassium hexahydroxoantimonate(V) (Sigma-Aldrich, St. Louis, MO, USA) to 950 mL of ultra-pure water and stirred overnight using a stir plate and stir bar. 50 mL of concentrated HNO_3 was added the following day to solubilize any remaining precipitant, bringing

the matrix to 5% HNO_3. Sb standards from 0.1 to 10 µg/L were prepared by serial dilutions of both Sb stock standards with 5 mM citric acid used as the diluent.

Elution orders of Cr and Sb species were determined by injecting 1 µg/L (ppb) individual standards of each species. Cr(III) elutes first at approximately 235 seconds and Cr(VI) elutes second at approximately 350 seconds. Sb(III) elutes first at approximately 75 seconds and Sb(V) elutes second at approximately 135 seconds. Sb speciation samples were allowed to sit at room temperature for 3 hours before analysis in order to ensure complete complexation of Sb with citric acid. All Cr samples and standards were allowed to sit at room temperature for 3 hours before analysis to ensure equilibrium is attained for the complexation of Cr(III) and EDTA.[8]

The coefficient of determination (R^2) was greater than 0.995 for each calibration curve of all Cr and Sb species. LOD and LOQ were calculated for each method using the formulas by multiplying 3.3 by the standard error of the y-intercept divided by the slope and multiplying 10 by the standard error of the y-intercept divided by the slope, respectively. Cr(III) and Cr(VI) LOD were 0.122 and 0.124 µg/L, respectively. Cr(III) and Cr(VI) LOQ were 0.370 and 0.376 µg/L, respectively. Sb(III) and Sb(V) LOD were 0.044 and 0.121 µg/L, respectively. Sb(III) and Sb(V) LOQ were 0.134 and 0.369 µg/L, respectively.

Working standards for each species in both analyses (Cr and Sb) were injected at 1 µg/L to verify instrument accuracy. All samples were injected in brackets of 10 samples followed by a carryover assessment blank, working standard injection, and a second carryover assessment blank. Carryover blank element concentrations were less than 10% of the concentration of the lowest calibration standard. All working standard injections recovered within 80 – 120% per analyte suggesting accurate results. Spike recoveries were conducted for both Cr species at 100 ppb in 50:50 PG:VG, with both analytes recovering between 97.5 – 100.2%. Initially, ELF Bar Flavored and Clear and Flum Pebble Flavored virgin e-liquids were used for the spike analysis, but complete reduction of Cr(VI) to Cr(III) (~100%) was observed despite analyzing the spiked samples immediately after dilution. Therefore, 50:50 PG:VG, free of flavorings, organic acids, and nicotine, was used for the spike analysis to reduce risk of rapid interconversion. Further studies are underway to investigate the cause of rapid interconversion in dPOD e-liquids. To evaluate the recovery accuracy of the LC-ICP-MS method for Sb, aerosol samples were

diluted 10-fold in 2% HNO3 and analyzed for total Sb by Q-ICP-MS. These results were then compared to the sum of Sb(III) and Sb(V) concentrations obtained from LC-ICP-MS. A mean Sb recovery of 82.7 ± 4.2% was achieved across all Flum Pebble aerosol samples, suggesting acceptable accuracy. However, LC-ICP-MS recoveries averaged 20% lower than Q-ICP-MS measurements, potentially due to Sb adherence to the LC vials in the absence of strong acid (HNO_3).

Aerosol samples were collected using a cold liquid trap, replacing the quartz wool collection apparatus previously employed for total metals analysis (**SI Section S1.5**). This modification was prompted by concerns that microscopic quartz fibers could damage the HPLC system and clog tubing, even after syringe filtration. Additionally, some anionic target analytes, such as Cr(VI) and Sb(V),[10,11] were expected to be repelled by the negatively charged quartz wool surfaces upon contact, potentially reducing capture efficiency. The cold liquid trap setup consisted of a 33 mm impinger PFA closure (Savillex, Eden Prairie, MN, USA) attached to a 50 mL polypropylene test tube filled with 20 mL of chilled (~5°C) sample diluent. PTFE tape was used to create an airtight seal between the impinger and the test tubes. The test tubes were placed in clean 250 mL beakers containing chilled (~5°C) ultra-pure water to maintain consistent cooling. Flow rates were manually adjusted to 1.7 L/min before each collection to ensure uniform sample collection. Air blanks were collected and analyzed using each method to assess potential background interferences affecting sample analysis. No elevated concentrations of Cr or Sb were detected in any of the air blanks analyzed.

Devices were fully charged and operated using vacuum lines modulated by solenoid valves with a time relay controller (PTR4-SP) for 100 puffs under standardized conditions: 2 seconds per puff, with a puff frequency of 2 puffs per minute (56.7 mL puff volume), replicating the puffing topography used in the total metals analysis (**SI Section S1.3**). After sampling, 5 mL of sample diluent was flushed through each of the two vacuum lines connected to the impinger closure to recover residual aerosol into the sample solution. The final sample volume of 30 mL and the total mass produced by the device were used to calculate species concentrations following the format of **Equation S3**.

Single ELF Bar and Flum Pebble Flavored and Clear devices were used for Cr speciation analysis with aerosol collections from 100 to 300 puffs. Triplicate Flum Pebble Flavored and Clear devices were

used for Sb analysis from 100 to 1200 puffs for the Flavored devices and 100 to 600 puffs for the Clear devices. Esco Bar devices were excluded from speciation analyses since the aerosol concentrations of Cr and Sb were deemed less hazardous compared to Pb, Ni, Cu, and Zn. Additionally, the high concentration of these non-target metals raised concerns about potential damage to the HPLC column and heavy dilutions used to reduce this risk would likely eliminate the possibility of quantitating Cr and Sb species.

S1.7. Cancer and Non-Cancer Risk Assessment Analyses

Inhalation risk assessment calculations followed recommendations by the U.S. Agency for Toxic Substances and Disease Registry (ATSDR) Guidance for Inhalation Exposure[12] and California Environmental Protection Agency (CalEPA) Office of Environmental Health Hazard Assessment (OEHHA) for Sb cancer risk.[13,14] Aerosol concentrations derived from **Equation S6** were used as the dose values in mg/m^3 for **Equations S7 – S9**. U.S. EPA's reference concentrations (RfCs: As[15], Sb(III)[16]), ATSDR's Minimal Risk levels (MRLs: Cd[17], Cr(III)[18]), OEHHA's Recommended Exposure Levels (RELs; Ni[19]), and U.S. EPA 's National Ambient Air Quality Standard for Pb[20] were used for health quotient (HQ) non-cancer risk assessment calculations (**Equations S7 – S8**). See **Table S15** for the reference values used for each element. U.S. EPA's published inhalation unit risk (IUR) value (m^3/mg) was used for Cd cancer risk assessment (**Equation S9**).[21] No EPA IUR's were available for Ni, As, and Pb cancer risk assessment, therefore OEHHA's IUR's were used instead.[22] Sb aerosol concentrations for Flum Pebble Flavored and Clear were adjusted by multiplying the total Sb concentrations by 33.5% and 5.2%, respectively, to account for the average relative abundances of Sb(III) (**Table S12**). No IUR exists for Sb(III), thus Sb(III) aerosol concentrations (mg/m^3) were multiplied by the inhalation rate 20 m^3/day to provide the concentrations in mg/day and divided by OEHHA's calculated Sb(III) No Significant Risk Level NSRL (mg/day) of 1.3×10^{-4} mg/day[13,14] (**Equations S10 – S11**) for comparison.

According to the U.S. Food and Drug Administration's (U.S. FDA) memorandum on the Use of Reference Values in the Toxicological Evaluation of Inhaled Tobacco Products (2019)[23], risk assessment calculations for tobacco products should adhere to the hierarchy of toxicity reference values with the EPA's Integrated Risk Information System reference values being the highest of three tiers, Provisional

Peer Reviewed Toxicity Values (PPRTVs) being the second tier, and CalEPA and ATSDR values as the lowest tier of reference values. Thus, in absence of EPA RfC's, MRL's were used (Cr(**III**), Cd) followed by REL (Ni). No RfC or MRL was available for Pb, therefore the NAAQS limit for Pb in ambient air was used as the non-cancer reference value for Pb.[20] This reference value was chosen over occupational risk reference values since the NAAQS limit is derived from estimates of general population exposures which accounts for sensitive populations.[20]

The U.S FDA Use of Reference Values in the Toxicological Evaluation of Inhaled Tobacco Products (2019) emphasizes that the use of occupational exposure limits for tobacco product risk assessments is inappropriate since these limits do not account for sensitive populations and should only be used if no values for the general population exist and the occupation limits are derived for the specific exposure.[23] In contrast to previous studies[24,25], we chose to avoid the use of oral risk limits and element doses in mg/kg/day for risk assessments. According to the ATSDR's Guidance for Inhalation Exposure, doses in mg/kg/day should be avoided for inhalation exposures unless there is simultaneous inhalation and oral exposure in which the contaminant targets the same organ or system through both routes.[12]

HQ < 1 predicts no potential for adverse non-cancer health effects, while HQ > 1 predicts potential for adverse non-cancer health effects at each dose (**Equations S7 – S8**). Cancer risk > 10^{-5} (1 in 100,000) indicates an unacceptable cancer risk at each dose. Cancer risk < 10^{-5} indicates an acceptable cancer risk at each dose (**Equation S9**).[26,27] Since the Sb(**III**) NSRL accounts for a cancer risk of 10^{-5} per $1.3x10^{-4}$ mg/day[13,14] we interpret values of **Equation S11** surpassing or equal to 1 as exceeding the cancer risk threshold of 10^{-5}, thus implying risk of cancer at a given dose.

$$Health\ Quotient\ (HQ) = \frac{Dose\ (mg/m^3)}{RFC,\ MRL,\ or\ REL\ (mg/m^3)} \qquad \text{(S7)}$$

$$Pb\ Health\ Quotient\ (HQ) = \frac{Pb\ Dose\ (mg/m^3)}{NAAQS\ Limit\ (mg/m^3)} \qquad \text{(S8)}$$

$$Cancer\ Risk\ = IUR\ \left(\frac{m^3}{mg}\right) x\ Dose\ \left(\frac{mg}{m^3}\right) \qquad \text{(S9)}$$

$$OEHHA\ Sb\ NSRL\ (mg/day) = \frac{10^{-5}\ x\ 70\ kg}{CSF\ (mg/kg*day)} \quad (S10)^{13}$$

$$Sb\ Cancer\ Risk\ Assessment = \frac{Dose\ (mg/day)}{NSRL\ (mg/day)} \quad (S11)$$

S1.8. Statistical Analyses

Mean element concentrations were calculated for each replicate sample per 100 puff intervals for 100 – 500 puffs. Linear regression analyses were performed on each element from 100 to 300 puffs and 100 to 500 puffs for the determination of linearity with respect to concentration over puff count. Two tailed t-tests with two sample unequal variance were performed on mean element concentrations between virgin and aged e-liquid data to assess the difference. Two tailed t-tests with two sample unequal variance were also performed on mean element concentrations in flavored and unflavored aerosols and e-liquids to assess the differences of flavoring-induced element emissions. Statistical significance (α) was set at 0.05. All statistical analyses were performed using Excel software (Microsoft).

Section 2: Supporting Results & Discussion

S2.1 Virgin E-Liquid Analysis

Virgin e-liquids were extracted from unused and 500-puff aged devices and analyzed for element content using Q-ICP-MS (**Figure S14, Table S2**). Elemental concentrations are reported in mass per mass (µg/kg); no aerosols were collected for these devices. Concentrations of Mn and Cd were higher in Esco Bar Flavored and Clear virgin e-liquids than in all other virgin e-liquids ranging from 27 to 200 µg/kg and 12 to 46 µg/kg, respectively (**Table S2**). Virgin e-liquid of an additional Esco Bar device, Esco Bar 6000 Puff Peach Watermelon was analyzed for elemental composition; no aerosols were collected. Pb, Ni, Cu, and Zn were detected in the virgin e-liquid at 17,200 ± 325, 1,420 ± 47, 61,600 ± 520, and 43,500 ± 726 µg/kg concentrations, respectively (**Table S2**). This finding suggests that the concentrations seen in the Esco Bar 2500 e-cigarettes are not specifically limited to Esco Bar 2500 devices and these metals might be widespread across many Esco Bar e-cigarette types and flavors at comparatively high

concentrations. Additionally, **Figures S5-S7** show strikingly similar designs between the Esco Bar 6000 and the Esco Bar 2500 devices, which may also further support the previous assumption.

S2.2 Full Aging Analyses (to 1500 puffs)

The ELF Bar flavored device had the longest lifespan at 1500 puffs (**Figure S10**). A sharp decline in aerosol mass produced was observed from 1400 – 1500 puffs, at which point the device ceased working. The Flum Pebble flavored device had the second longest lifespan at 1400 puffs (**Figure S10)**. A steady decline in aerosol mass production was observed from 1000 – 1400 puffs, at which point the device no longer functioned. The ELF Bar clear device had the shortest lifespan at 1300 puffs, steadily losing aerosol mass production from 900 – 1300 puffs until complete loss of function (**Figure S10**).

The decrease in concentrations seen between 400-600 puffs, (**Figure 10**), for Cr and Ni below could be explained by the time between collections. Aerosol samples were collected in sets of 200 – 300 puffs per day for the first 500 puffs and were given weeklong intervals before continuing the remainder of collections. Elements may have accumulated in the wick near the coil upon use and diffused away from the coil to the rest of the wick during the weeklong rests, decreasing metal concentrations in the 600 puff aerosols. This sampling strategy was done to ensure consistent 100 – 500 puff collections on all replicates; however, the effects on element concentrations were not apparent until after data analysis.

In replicates of all ENDS analyzed from 100 to 500 puffs (n = 3), elements not identified in any metallic components (As, Se, Cd, Sn, Mn, Ba, and U) were often measured at low concentrations or below the limits of detection or quantitation and did not vary with device use or puff count suggesting a lack of emissions with respect to aging (**Tables S1, S3**). Ni, Cu, Zn, and Pb concentrations in Esco Bar (Flavored and Clear) aerosols were highest from 100 to 200 puffs (274 to 8,930 µg/kg Ni, 4,450 to 24,100 µg/kg Cu, 13,100 to 87,500 µg/kg Zn, and 3,850 to 51,900 µg/kg Pb, **Table S3**) and decreased afterward from 300 to 500 puffs (**Table S3**) likely due to the inability to recharge Esco Bar 2500 devices. The aerosol concentrations measured here represent significant risk to the user, especially in the case of Pb (as high as 51,900 µg/kg) implying that in a single set of 100 puffs an Esco Bar user may be significantly exposed to Pb.

Sb concentrations in aerosols across all Flum Pebble (Flavored and Clear) aerosol samples increased linearly from 100 to 500 puffs (R^2 = 0.832 to 0.894, 116 to 664 µg/kg Sb) (**Tables S3**, **S4**). A single replicate of ELF Bar Flavored 0% nicotine (replicate 1) increasingly emitted 97 to 271 µg/kg Sb in aerosols from 100 to 500 puffs (**Table S3**) whereas the other two replicates (2 and 3) emitted an estimated 1 to 7 µg/kg Sb in aerosols. Since Sb was not identified in any of the metal alloys but present in virgin e-liquids, the concentrations observed in these aerosols implies the transfer of Sb from the e-liquid to the aerosols and risk of exposure to toxic elements contaminating e-liquids.

S2.3 Aged e-liquid & 500-puff Aerosol Analysis

When comparing aged e-liquids to their respective 500-puff aerosols, Cr and Ni concentrations in the 500-puff aged e-liquids and 500-puff aerosols for ELF Bar and Flum Pebble devices were elevated suggesting the leaching of these metals from a source within the device to the aerosol (**Figure S15, Tables S7.1 – S7.2**). **Figure S15** plots the concentrations of Cr and Ni in the 500-puff aerosols over the 500-puff e-liquids to assess linear correlations. The coefficient of determination between data points for both metals is 0.921 (**Figure S15**), which shows a strong correlation between aerosol and aged e-liquid concentrations. This strongly supports our hypothesis of a direct causal relationship between increased metal concentration with respect to device aging potentially as a result of coil degradation. Linear regression analysis was also performed on aged e-liquid and 500-puff aerosol concentrations for Cu, Zn, As, Cd, Sn, Sb, Bi, and Pb. However, most 500-puff aerosol concentrations for these elements were below the method's LOD and LOQ, and were therefore excluded from the statistical analysis.

Esco Bar Ni maximum aerosol concentrations were 2,190 – 2,300 µg/kg, representing approximately 7 – 10% of the Ni measured in their respective virgin e-liquids which suggests difficulty in transfer of Ni from the e-liquid to the aerosols (**Tables S2 – S3**). Aerosol concentrations of Cu, Zn, Sb, Pb, and Bi also never surpassed their concentrations in both flavored and unflavored Esco Bar e-liquids. Pb aerosol concentrations were measured at 29.2% of the aged e-liquid Pb concentrations, which was the highest transfer of metal to the aerosol amongst the highly concentrated elements in these e-liquids. An additional hypothesis could be that since these metals do not directly originate from the coil, there is less

opportunity for the metals to be transported from the e-liquid to the aerosol droplets. Metals from the coil may leach at higher concentrations directly into the aerosol. In a study by Rastain et al. (2022)[28] using a 3rd generation ENDS (Vaporesso Revenger Mini), Cr and Ni transferred preferentially to the aerosols rather than the e-liquid whereas Cu and Pb preferred to remain in the tank e-liquid rather than aerosols. These results suggest differences in element transfer to aerosols, which may be a result of the source of element and proximity to the aerosol (i.e. heating coils) or the properties of the element in e-liquids.

Aged e-liquid element concentration differences were assessed between flavorings and nicotine containing devices (ELF Bar Flavored and ELF Bar 0% Nicotine Flavored) (**Figure S14, Tables S6.1-S6.2**). Significant differences were observed between aged e-liquids of ELF Bar flavorings for Cr, Ni, Zn, and Sn, Flum Pebble flavorings for Cr, Fe, Ni, Cu, As, and Sb, and Esco Bar flavorings for Cr, Mn, Fe, Ni, Cu, Zn, Cd, Sn, Sb, Bi, and Pb (**Table S6.2**). Significant differences were also observed between ELF Bar nicotine and nicotine-free aged e-liquids for Fe, Ni, and Zn (**Table S6.2**). Given that some elements significantly increased between virgin and aged e-liquids, these could be additional aging effects caused by flavorings and nicotine. However, the results of this statistical analysis should be interpreted with caution due to a lack of statistical power across virgin and aged e-liquids.

S2.4 Aerosol Element Emission Comparisons

Element mass emissions in aerosols from each device from 100 – 500 puffs were summed and compared to one another to assess which devices produced the most element emissions (**Table S5.1**). Statistical analyses were conducted on mass emissions to determine differences in emissions by flavors and the presence of nicotine (**Table S5.2**). ELF Bar devices, as a whole, emitted the greatest Cr mass of any device type and Flum Pebbles produced the most Sb of all device types (**Table S5.1**). Esco Bar devices emitted the greatest mass of Pb, Ni, Cu, Zn, Cd, Sn, and Bi, relative to the other devices (**Table S5.1**).

No significant differences were observed in element emissions between ELF Bar flavors and nicotine-containing devices (**Table S5.2**). The results of this statistical analysis mostly disagree with the results of aged e-liquid flavorings and nicotine comparisons, discussed in **SI Section S2.2**, with the exception of the

Flum Pebble Cr and Ni aerosol mass differences. However, the small samples sizes of both assessments hinder the formation of any strong conclusions. Thus, the impact of flavorings and nicotine on element emissions presented here should be carefully considered. More studies with larger sample sizes are needed to accurately define these effects.

Contamination with Cu, Zn, and Pb was identified in six aerosol samples, including the following: ELF Bar Clear (Replicate 1, 200 puffs), ELF Bar 0% Nicotine (Replicate 3, 100 puffs), Flum Pebble Flavored (Replicate 2, 300 puffs), and Flum Pebble Clear (Replicate 1, 100 puffs; Replicate 2, 200 and 400 puffs) (**Table S3**). The simultaneous presence of Cu, Zn, and Pb, representing an alloy, in each of these six samples suggests a similar contamination source. Upon analytical investigation, it was determined that the metal solenoids used to puff the devices were the source. This contamination was observed in only 6 out of 131 total samples, indicating it was a rare occurrence. Importantly, Esco Bar virgin e-liquids consistently exhibited concentrations of these elements more than 1,000-fold higher than those of the affected samples, ruling out contamination as the cause of the extreme results observed with this brand. Cu, Zn, and Pb concentrations from the contaminated samples were excluded from mean calculations for each device to ensure accuracy.

In comparison to aerosol values in literature, Esco Bar devices emitted Pb in aerosols as high as 51,900 µg/kg of in a single set of 100 puffs (**Table S3**), which surpasses the maximum Pb concentration reported by Zhao et al. 2019 (Table 2, OD2, 40W, Q3) of 4141 µg/kg by approximately 14-fold.[2] Thus underscoring the elevated risk of Pb exposure from Esco Bars relative to some of the highest Pb concentrations reported in e-cigarette aerosols. The differences between this study and others[2,3,29] could be attributed to inconsistencies between puffing topographies (puff volume, puff duration and puff count). An attempt was made to compare the mass per volume of air (mg/m^3) aerosol concentrations provided in this study to those in Ahererra et al. 2023, but the calculations for our studies differed due to the volumes of air used to normalize element mass (mg) to volume of air (m^3). Therefore, we opted to compare our mass per mass (µg/kg) values to those of the same units provided in the supplementary information of Ahererra et al. 2023.[3]

In comparison to traditional combustion cigarettes, a single pack of cigarettes could expose a user to between 0.97 – 2.64 µg of Pb.[30] Mean cumulative Pb masses in Esco Bar aerosols from 100 – 200 puffs were quantified at 14.2 ± 1.3 µg in the Flavored aerosols and 43.6 ± 5.6 µg in the Clear aerosols, suggesting extensive Pb exposure over the lifespan of these devices with use (**Table S13.2**). In a study by Pappas et al. 2014, 50 cigarette brands available in the US were puffed using the Health Canada intense smoking regimen (55 mL puff volume at 2 puffs per minute) to determine metal contents in mainstream smoke including Pb.[31] When comparing the masses of Pb emitted in all individual Esco Bar 100 puff collections to the highest mass produced by a cigarette (Benson & Hedges Green 100s HP Menthol)[31], a single set of 100 puffs from Esco Bar devices could expose a user to an amount of Pb equivalent to consuming 19 packs of cigarettes in a day (**Table S13.3**). Cr and Ni e-cigarette emissions were also vastly higher than maximum Cr and Ni masses reported in the intense smoking regimen for combustion cigarettes (**Table S13.3**). With concentrations of the Esco Bar dPODs being vastly higher than ENDS aerosols analyzed in previous studies[2,3,29], this is of great concern.

This analysis reveals that modern disposable e-cigarettes emit more metals and metalloids in their aerosols than older ENDS, suggesting a higher potential for metal and metalloid exposure. The comparisons made here may underestimate full comparative metal and metalloid exposure across ENDS, particularly across the extended lifespan of dPODs and the lack of an open system to exchange worn coils and refresh contaminated e-liquids.

S2.5 LC-ICP-MS Cr & Sb Speciation

A single ELF Bar Flavored and Clear each, and a Flum Pebble Flavored were puffed from 100 to 300 puffs to determine the presence and concentration of carcinogenic Cr(VI)[18] in aerosols. No Cr(VI) peaks (retention time: 350 seconds; main text **Figure 4A**) were detected in all aerosol samples analyzed. Non-toxic Cr(III) (retention time: 235 seconds; main text **Figure 4A**) was the exclusive Cr species present in all aerosol samples analyzed. Therefore, we conclude that Cr(III) is the only species of Cr emitted from the e-cigarettes analyzed in our study. Cr(VI) is a powerful oxidizing agent at low pH, so the absence of Cr(VI) in aerosols was not unexpected given the combination of heat, acidic e-liquids (pH ~ 4.5; **Table S17**), and

the presence of potential reducing agents such as benzoic acid and flavorings in the e-liquids.[10] However, a recent study in 2024 identified the presence of Cr(VI) in aerosols produced by an open-system e-cigarette device (3rd generation; VOOPOO Drag X) at the highest power tested (60W). Approximately 15% of the total Cr was detected as Cr(VI), showing that Cr(VI) may be present in e-cigarette aerosols.[32] Further analyses are needed to determine which e-liquid and device conditions are most suitable for the production of Cr(VI) in aerosols and to what extent.

Triplicate Flum Pebble Flavored and Clear devices were puffed from 100 to 1200 puffs and 100 to 600 puffs, respectively, and analyzed for Sb speciation. Maximum puffs of 600 and 1200 were chosen for Clear and Flavored devices, respectively, to best represent puff ranges used in the risk assessment analysis per each flavor and assess speciation trends over device lifespans (**SI Section S2.6**). Mixtures of Sb(III) (retention time: 75 seconds; main text **Figure 4B**), and Sb(V) (retention time: 135 seconds; main text **Figure 4B**) were observed across most Flum Pebble Flavored and Clear aerosols. Relative abundance of Sb(III) was calculated as the concentration of Sb(III) (μg/kg) divided by the sum of Sb(III) (μg/kg) and Sb(V) (μg/kg) multiplied by 100% (**Table S14**). Mean % Sb(III) across all Flavored aerosols was 33.5 ± 19.1%, ranging from 4.0 to 74.4% (**Table S14**), whereas mean % Sb(III) across all Clear aerosols was 5.2 ± 3.5%, ranging from 0.0 to 13.1% (**Table S14**). The results of the Sb speciation analysis revealed two important findings: (1) carcinogenic Sb(III)[33–35] was present in Flum Pebble Flavored and Clear aerosols as high as 74.4% of the relative abundance (**Table S14**) and (2) Flum Pebble Flavored aerosols produced on average 6 times more Sb(III) than the Clear aerosols (**Table S14**). A detailed mechanistic analysis is beyond the scope of this experiment. Future studies are needed to clarify the mechanisms underlying the differences in Sb(III) production between Flum Pebble Flavored and Clear aerosols.

S2.6 Cancer and Non-Cancer Risk Assessment Analyses

Mean Ni cancer risk values of ELF Bar Flavored and Clear exceeded the cancer risk limit of 10^{-5} between 200 to 500 puffs with risk values ranging from 1.00×10^{-5} to 1.23×10^{-5} (**Figure S23A**, **Table S16).** Esco Bar Flavored and Clear mean Ni cancer risk values also exceeded the risk limit of 10^{-5} between 100 to 300 puffs (1.19×10^{-5} to 4.12×10^{-5}; **Figure S23A**, **Table S16**). In the full aging cancer

risk analysis, ELF Bar Flavored, ELF Bar Clear, and Flum Pebble Flavored Ni cancer risk values surpassed the cancer risk limit between 400 to 1500 puffs, 1000 to 1300 puffs, and 1000 puffs, respectively. ELF Bar Clear Ni values were highest of the three devices ranging from 1.30 x 10^{-5} to 1.06 followed by ELF Flavored (1.20 x 10^{-5} to 1.02 x 10^{-4}), and Flum Pebble Flavored (1.61 x 10^{-5}) (**Figure S23C**, **Table S16**).

Antimony (Sb) cancer risk values only exceeded the NSRL of 1.3 x 10^{-4} for the Esco Bar Flavored aerosols (100 to 300 puffs) (**Figure S23B, Table S16**). However, Esco Bar Sb aerosol concentrations were not adjusted to account for the relative abundance of Sb(III) and thus these values are likely overestimates of Sb(III) exposure risk. See **Section S1.6** for more information. The fully aged Flum Pebble Sb cancer risk values exceeded the NSRL as early as 200 puffs until 1200 puffs and ranged from 1.29 to 4.28 in an increasing manner, suggesting increasing risk of cancer from Sb(III) exposure over the device life cycle (**Figure S23D, Table S16**). Sb non-cancer risk values did not surpass the HQ, implying little to no risk of Sb non-cancer effects.

Excessive non-cancer risk values for Ni were observed often across ELF Bars, Esco Bars, and the fully aged Flum Pebble aerosols. ELF Bar Ni non-cancer risk values from 100 to 500 puffs almost always surpassed the health quotient (HQ = 1) (1.00 to 3.38**; Figure S24A, Table S16**). The fully aged ELF Bars Flavored and Clear presented the largest Ni non-cancer risk values spanning from 1.02 to 29.2 in an increasing fashion (**Figure S24C, Table S16**). The fully aged Flum Pebble exhibited Ni non-cancer risk between 900 to 1100 puffs (1.33 to 4.41; **Figure S24C, Table S16**). Cr(III) non-cancer risk exceeded the HQ in a single instance across all samples in the fully aged ELF Bar Clear at 1200 puffs (1.66; **Figure S24D, Table S16**).

Lead (Pb) non-cancer risk values only surpassed the HQ with Esco Bar aerosols from 100 to 300 puffs ranging from 1.24 to 4.25; (**Figure S24B, Table 16**), which is unsurprising given the immense Pb aerosol concentrations measured in these devices. On the other hand, Pb did not surpass cancer risk limits in the cancer risk assessment. According to the ATSDR Toxicological Profile for Lead, there is limited evidence of Pb carcinogenicity and thus, the Pb cancer risk values appear logical.[36] As and Cd risk values did not surpass non-cancer and cancer risk limits (**Table 16**).

Decreases in cancer risk for ELF Bar Clear (1200 to 1300 puffs) and Flum Pebble Flavored (1000 to 1400 puffs) (**Figures S23C,D – S24C,D**) attributed to loss of device power and functionality, highlighted in **Figure S10**. This data does not directly suggest diminishing cancer risk towards the end of the device life cycle. Less aerosol generation may result in a user taking more puffs, thus a summation of the metal concentrations and risk between 1200 to 1300 puffs (ELF Bar Clear) and 1000 to 1400 puffs (Flum Pebble Flavored) may be more appropriate.

A separate study on dPOD metal emissions by Lin et al. (2022) also concluded cancer risk for Cr and Ni at the aerosol concentrations they measured.[24] Fowles et al. compiled e-liquid and aerosol metals data reported by various studies and performed an depth risk assessment reaching similar conclusions of non-cancer and cancer risks for Cr, Ni, and Pb concentrations found in previous studies.[2,25,29] In our analysis, we chose to omit Cr from cancer risk assessment since we did not identify Cr(VI) in any of the aerosol samples measured for Cr speciation. Cr(III) is considered an essential metal to some but the topic has sparked debate in recent years.[37,38] Essential metals may still be toxic, particularly in the case of excessive exposure.[39]

Section 3: Supplementary Figures

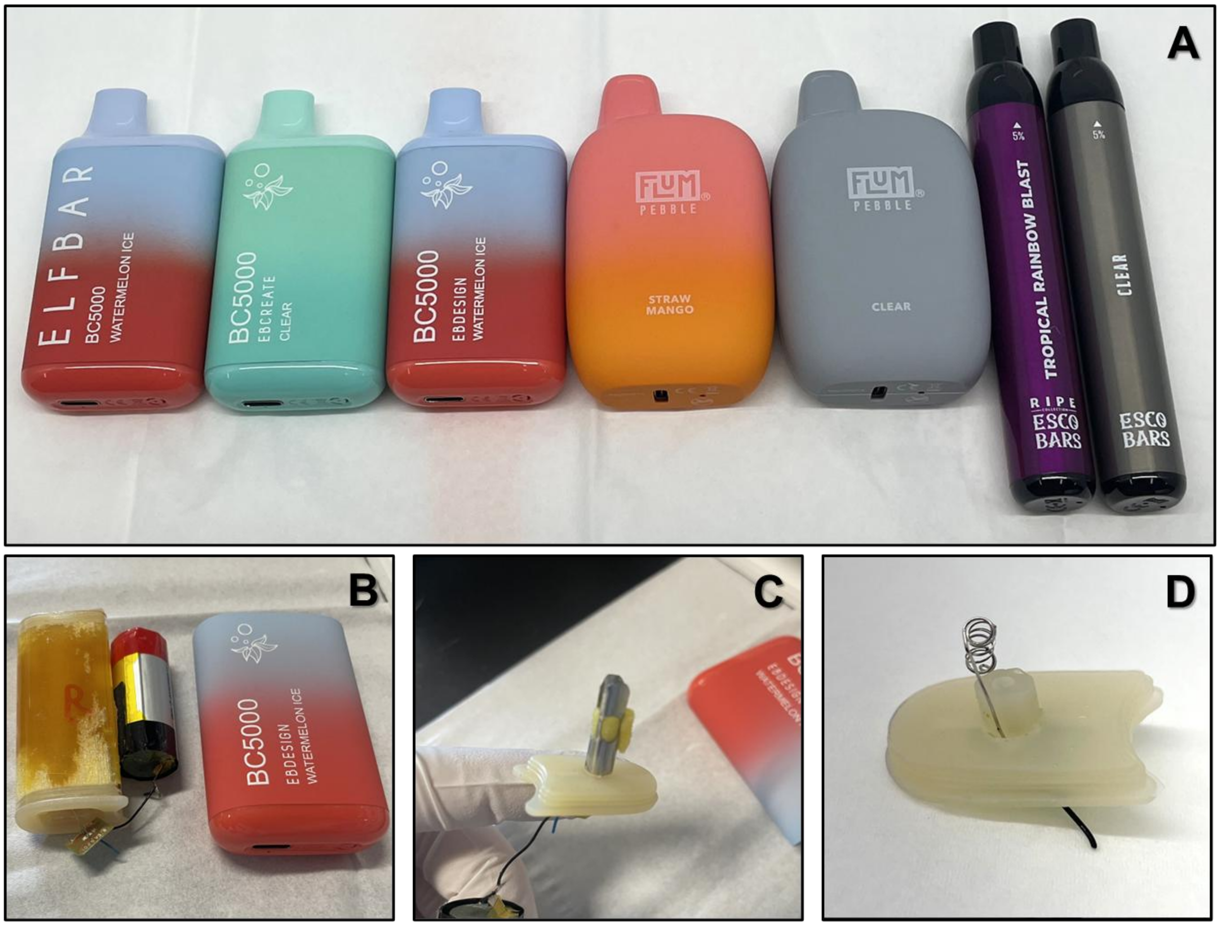

Figure S1: Collection of the seven disposable POD e-cigarettes (dPODs) or electronic nicotine delivery systems (ENDS) analyzed in this study and disassembly of an ELF Bar flavored device. (A) Devices displayed in the following order from left to right: ELF Bar BC5000 Watermelon Ice, ELF Bar (“EBCreate”) BC5000 Clear, ELF Bar (“EBDesign”) BC5000 Watermelon Ice 0% Nicotine, Flum Pebble 6000 Straw Mango, Flum Pebble 6000 Clear, Esco Bar 2500 Tropical Rainbow Blast, and Esco Bar 2500 Clear. ELF Bar manufacturer iMiracle Shenzen has been accused of changing the name of ELF Bar products to EBCreate and EBDesign to skirt an FDA import ban in 2023.[40,41] (B) Battery attached to computer chip and atomizer enveloped in a wick containing e-liquid. (C) Metal “sheath” holding separate smaller pieces of wick to the coil. (D) Wire coil separated from computer chip and other components by a plastic holder.

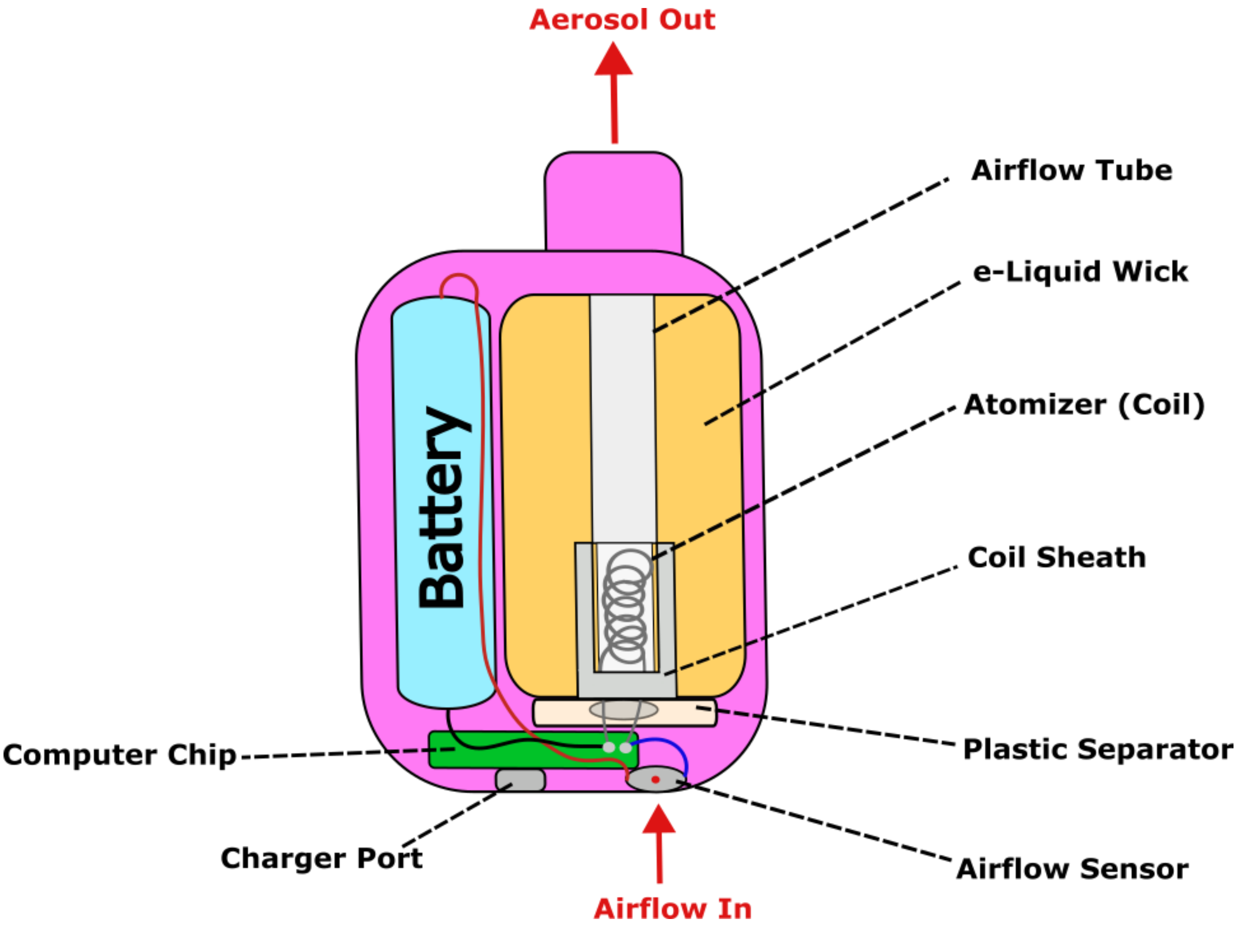

Figure S2: Simplified diagram of basic dPOD internal components. Airflow tubes help funnel and deliver aerosol to the mouthpiece for inhalation. E-liquid wicks contain the e-liquid within the device acting as a reservoir. Coils are surrounded by a metal sheath, which is believed to hold the coil and smaller pieces of e-liquid-soaked wick to the coil for proper aerosolization of the e-liquid. Coils, made of wire or mesh, are in line with the airflow tube to deliver aerosol upon activation and heating. In the case of ELF Bar and Flum Pebble devices, all aerosol delivering components (i.e., airflow tubes, wicks, coils, and sheaths) are separated from the rest of the internal components such as the battery, computer chips, and airflow sensors by a plastic separator of varying complexity depending on the brand (**Figures S3 – S4**). Esco Bar design slightly differs with the use of battery connectors to direct current to the coil rather than a computer chip (**Figure S5)**. Airflow sensors, located on the bottom of the device alongside openings for air intake, are responsible for activating the battery circuit to power the coil and produce aerosol as air is sent through the device to the mouthpiece. Airflow must be high enough for the sensor to activate the circuit.

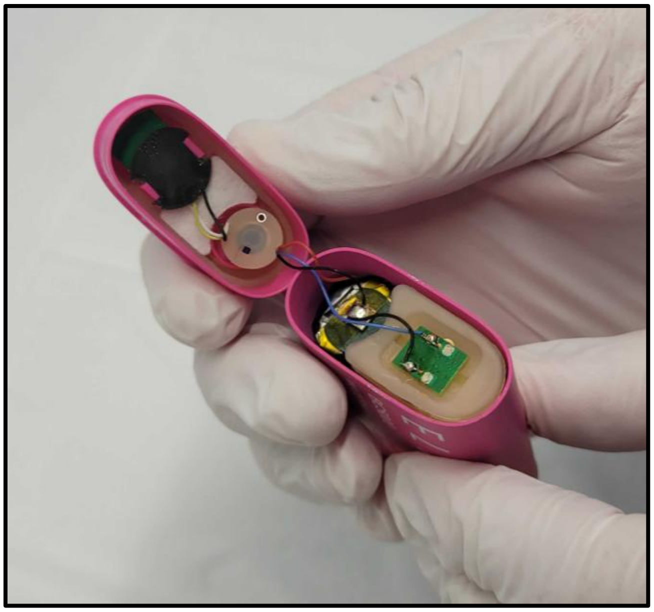

Figure S3: Display of ELF Bar Flavored airflow sensor, encapsulated by light grey plastic shown in the top piece, and computer chip wiring. The openings seen within the airflow sensor construct are possibly air intake openings. It is unclear how the air entering the device flows through to deliver aerosol since there appears to be no direct path for the air to travel through the device.

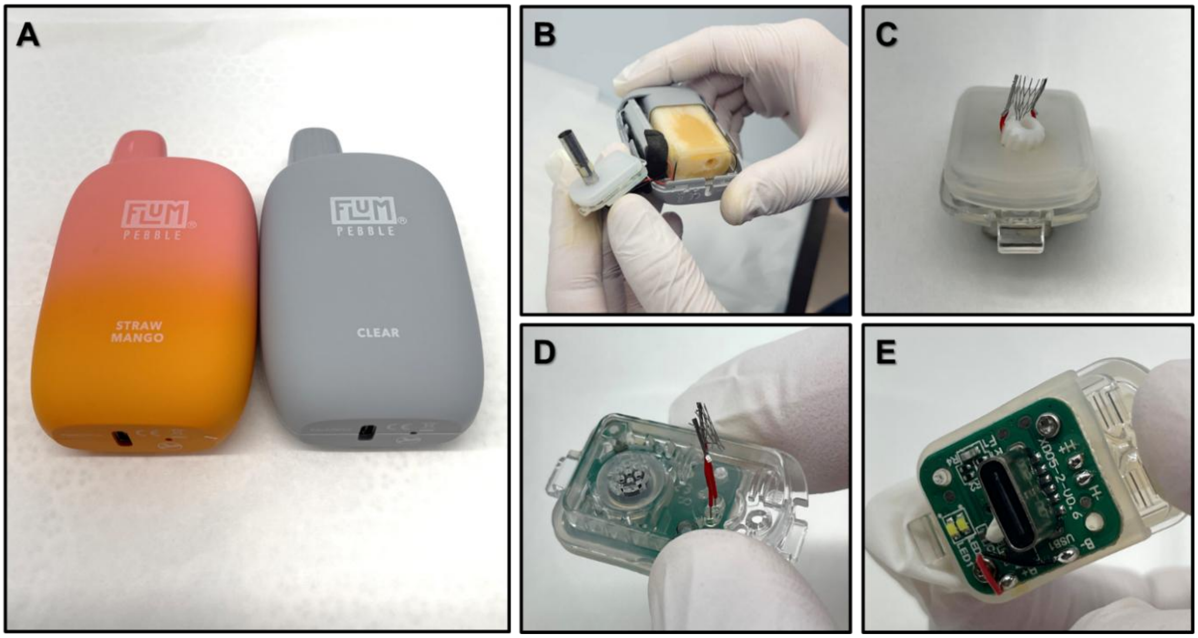

Figure S4: Display of Flum Pebble devices and disassembly of a Flum Pebble 6000 Clear device. (A) Flum Pebble 6000 Straw Mango (left) and Clear (right) devices. (B) Coil and sheath removed from the wick of a virgin Flum Pebble Clear. (C) Flum Pebbles utilize mesh coils for aerosolization of e-liquid. (D) Top view of the computer chip housing and without the plastic housing. The openings cut into the plastic to the left of the coil are thought to be part of the airflow sensor. (E) Charging port located on the bottom of the device. Flum Pebbles appear to use the most complicated plastic separation units of these three dPOD brands.

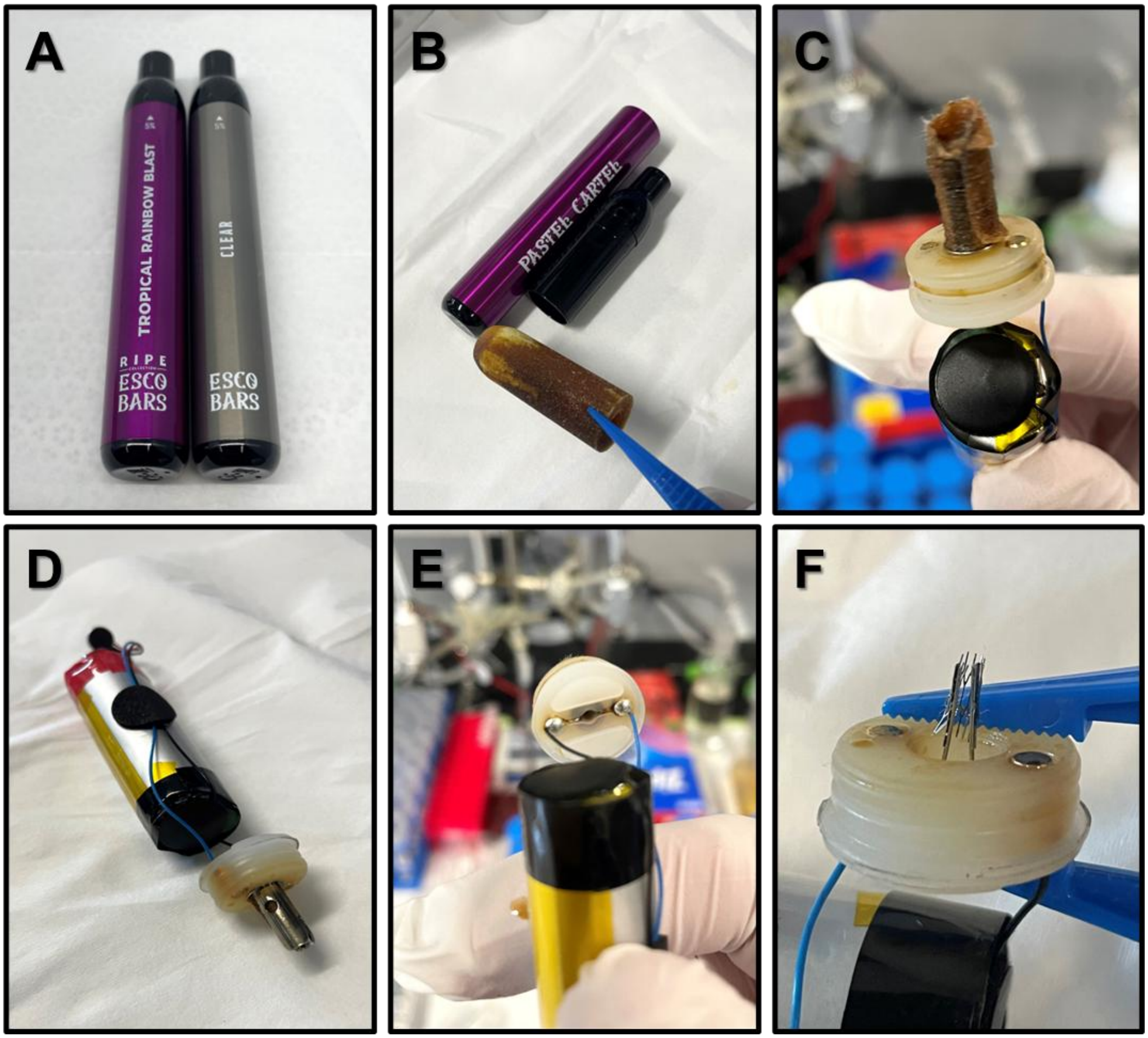

Figure S5: Display of Esco Bar e-cigarettes and disassembly of an Esco Bar 2500 Tropical Rainbow device. (A) Esco Bar Tropical Rainbow Blast and Clear devices. (B) Wick of a virgin Esco Bar flavored device. (C) Additional piece of wick wrapped around the coil sheath. (D) Battery, plastic separator, and airflow sensor (shown above the red tape on the battery). (E) Bottom of the plastic separator showing the bottom ends of battery connectors used to directly deliver power to the coil. Wires from the battery and airflow sensor are soldered here. (F) Esco Bar devices utilize mesh coils for aerosolization of e-liquid. Battery connector tops, metallic units seen to the right and left of the coil, are in direct contact with the e-liquid wick.

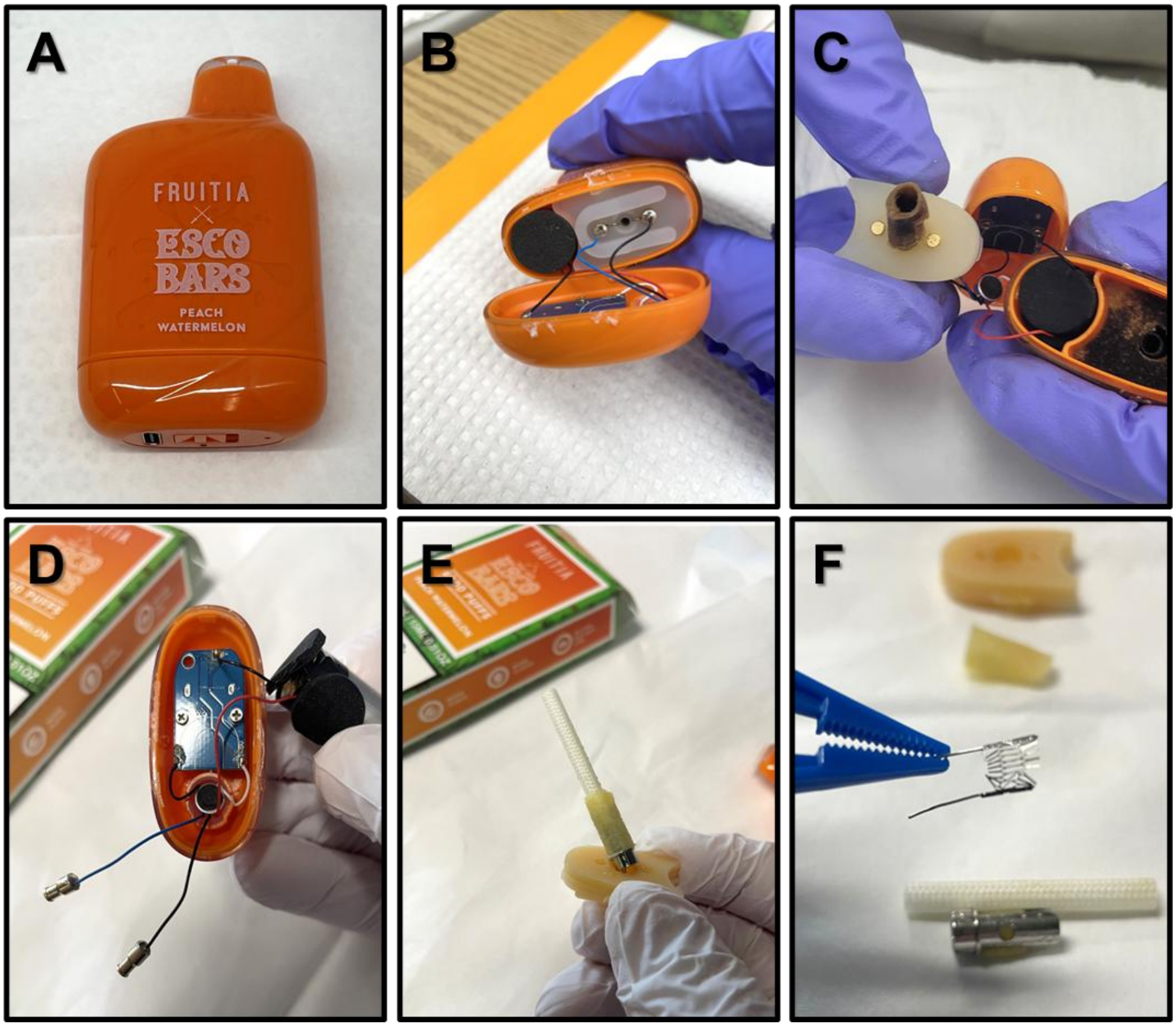

Figure S6: Display and disassembly of an Esco Bar 6000 flavored device. (A) Esco Bar 6000 Peach Watermelon device. (B) Bottom of the plastic separator showing the bottom ends of battery connectors used to directly deliver power to the coil. (C) Additional piece of wick wrapped around the coil sheath. Battery connector tops, metallic units seen to the left and right of the wick-wrapped coil, are in direct contact with the wick, seen on the far right still held within the rest of the device casing. (D) Full battery connectors removed from plastic separator, computer chip, and airflow sensor seen directly below the computer chip. (E) Airflow tube attached to additional wick and coil sheath. (F) Esco Bar 6000 devices also utilize mesh coils for aerosolization of e-liquid.

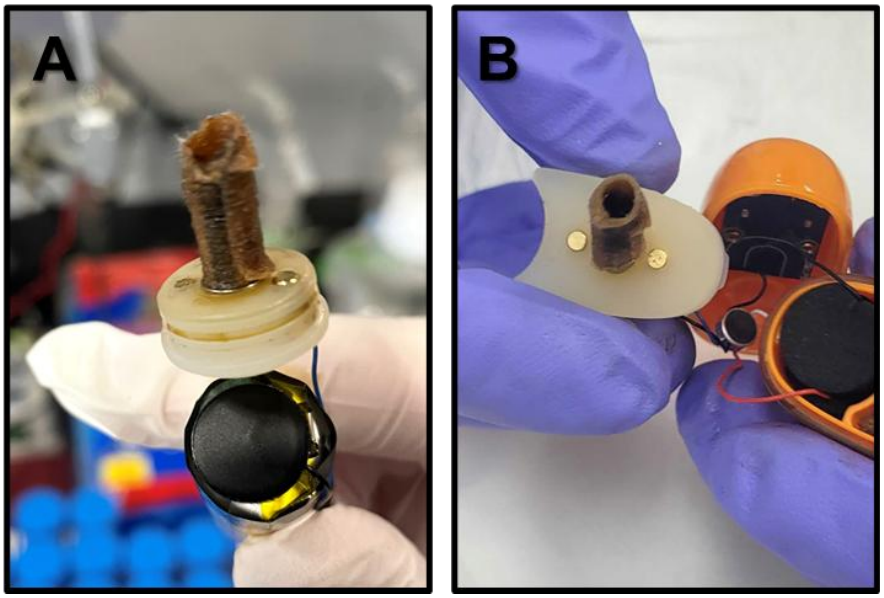

Figure S7: Comparison of Esco Bar 2500 and Esco Bar 6000 designs. (A) Esco Bar 2500 Tropical Rainbow Blast. (B) Esco Bar 6000 Peach Watermelon. Both types of Esco Bar devices appear to utilize nearly identical designs for modulating coil activation by using battery connectors, directly exposed to e-liquid, to power the coil.

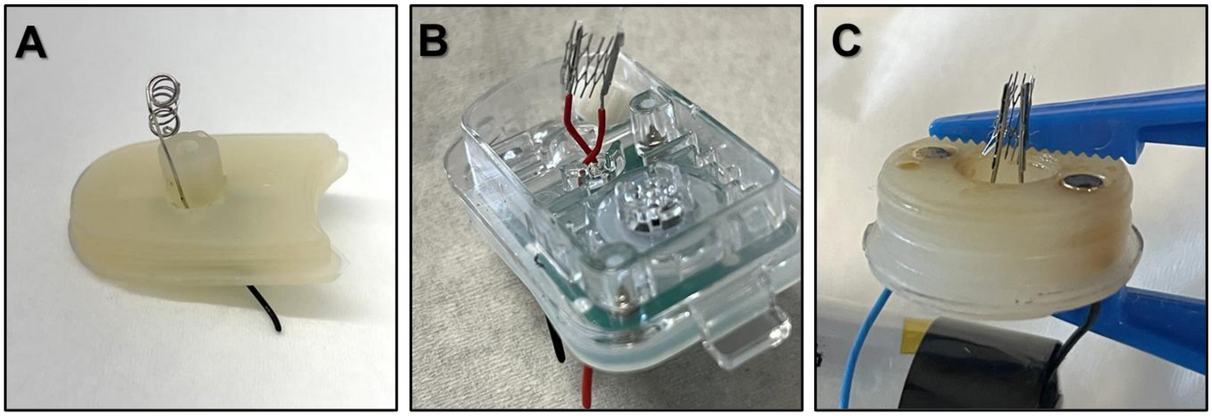

Figure S8: Coils analyzed by LA-ICP-MS. (A) ELF Bar wire coil removed from ELF Bar BC5000 Watermelon Ice device. (B) Flum Pebble mesh coil removed from Flum Pebble 6000 Straw Mango device. (C) Esco Bar mesh coil removed from Esco Bar 2500 Tropical Rainbow Blast device.

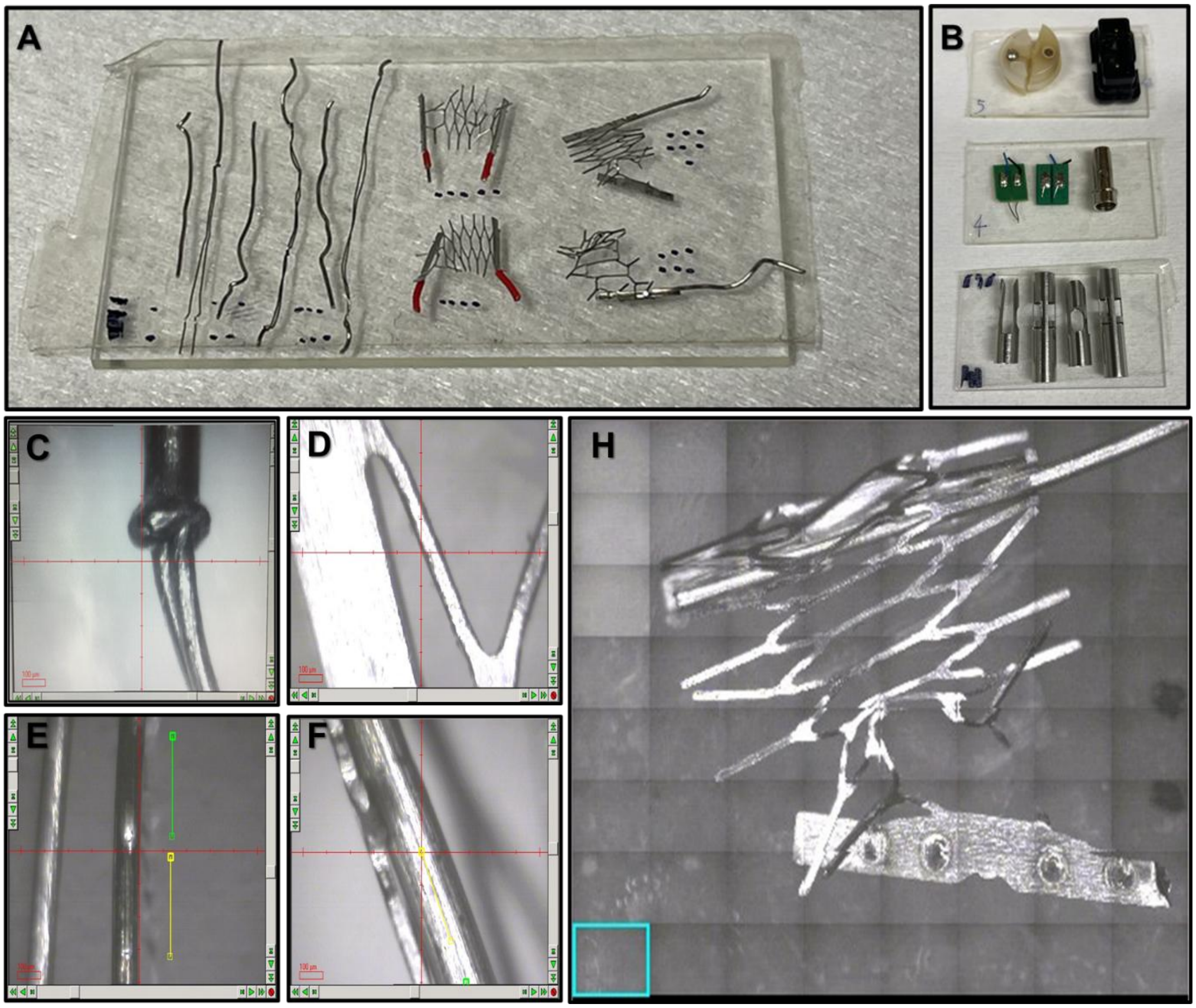

Figure S9: Samples prepared for laser ablation by placing coils and components on glass slides and flattened. Slides were inserted into the laser ablation sample holder and analyzed. (A) Virgin coils with ELF Bar wire coils on the left, Flum Pebble mesh coils in the middle, and Esco Bar mesh coils on the right. (B) Slide 5 (top): Esco Bar battery connectors attached to plastic wick and coil holder on the left (cut in half to analyze both sides of the connector) and an additional sample analyzed for a separate study. Slide 4 (middle): ELF Bar flavored chip facing upward on the left, ELF Bar clear chip facing downward in middle, and Esco Bar flavored sheath on the right. Slide 3 (bottom) in order of left to right: ELF Bar flavored sheath, ELF Bar clear sheath, Flum Pebble flavored sheath, and Flum Pebble clear sheath. (C): Laser ablation high zoom image of ELF Bar flavored wire coil. Wire coils contained two separate inner wires likely used as the heating element and a thicker wire coating surrounding the rest of the wire, seen at the top of the image. (D): Flum Pebble mesh coil under high magnification. (E) ELF Bar heating element wires with linear ablation paths shown with green and yellow markers. (F): Flum Pebble mesh coil support image under high magnification.(H) Mapping image of Esco Bar flavored mesh coil. (C-H): Each tick mark represents 100 μm.

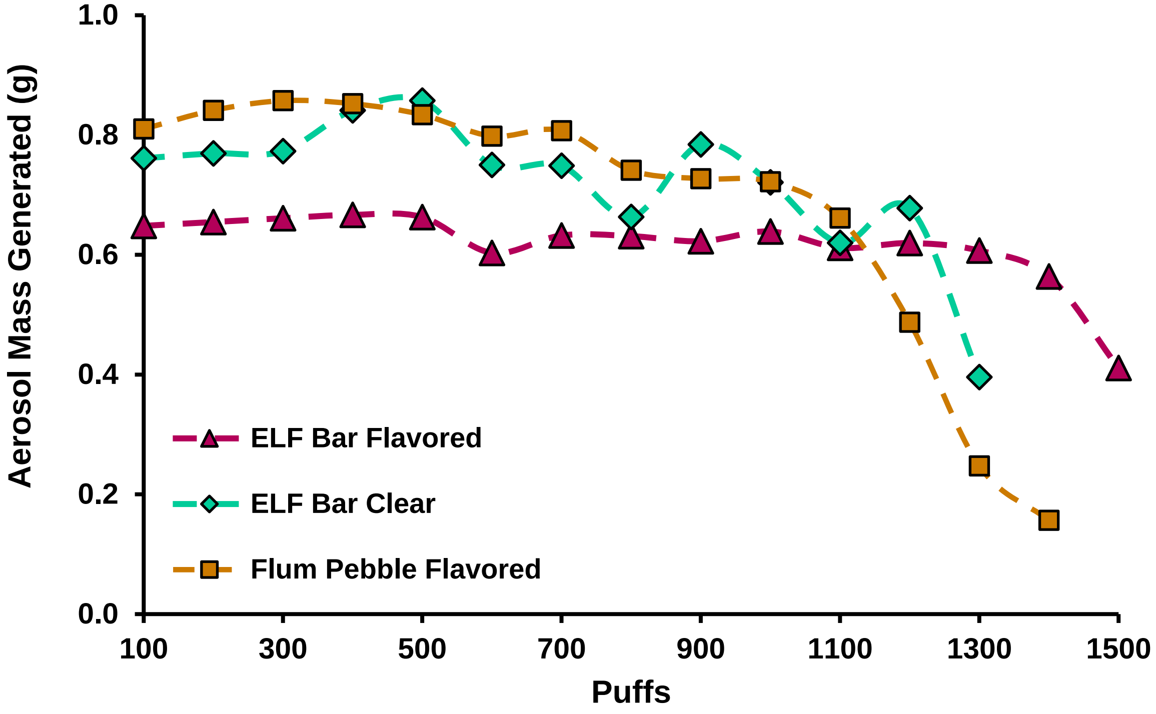

Figure S10: Aerosol mass generation (g) of ELF Bar Flavored (magenta), ELF Bar Clear (sea green), and Flum Pebble Flavored (orange) devices, each puffed in 100 puff intervals until the complete loss of device function. Loss of function, denoted by decreasing mass generation, is seen within the last 500 puffs of each device.

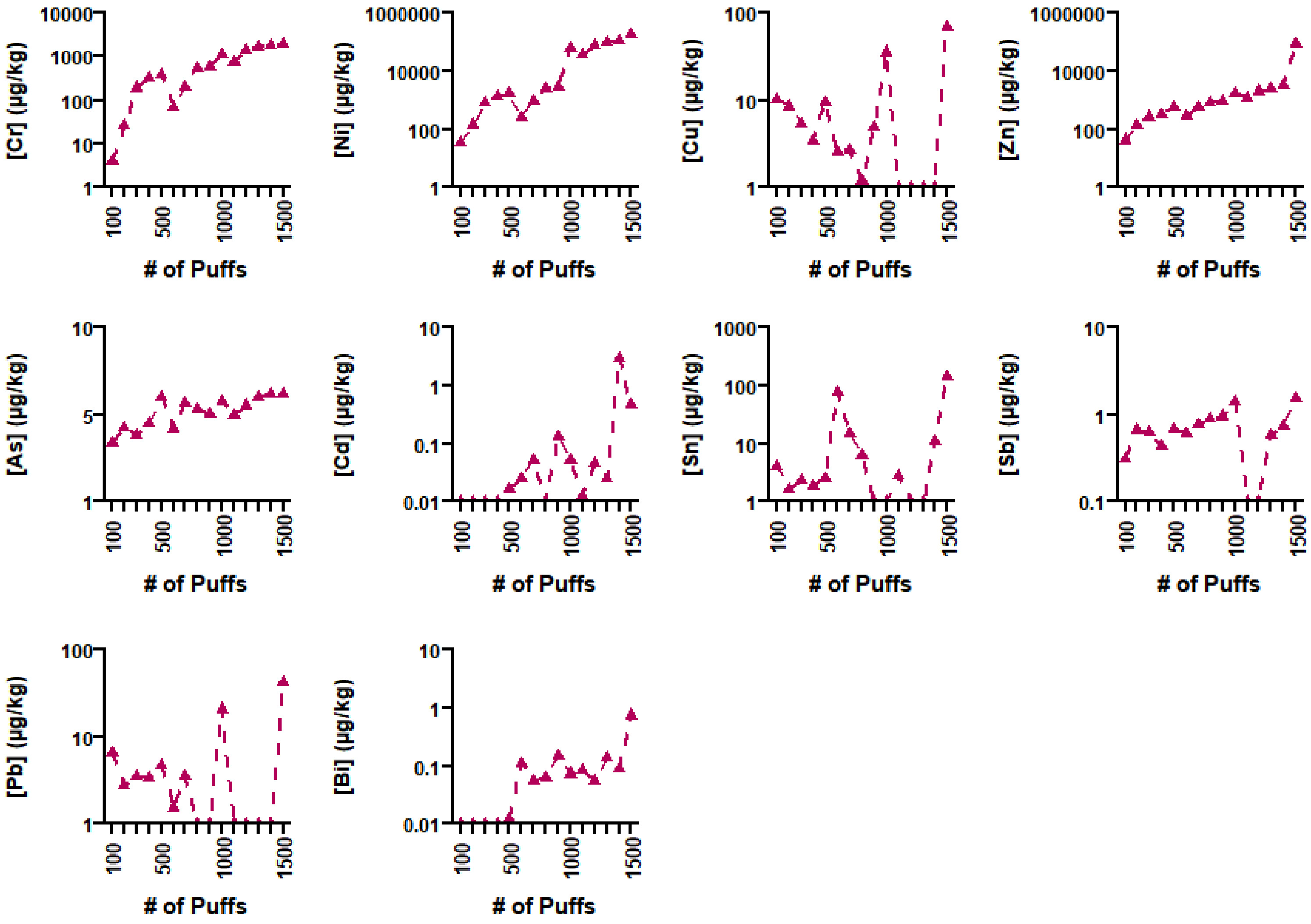

Figure S11: ELF Bar Flavored concentrations (µg/kg) from full aging analysis (100-1500 puffs) (*n* = 1). See **Table S3** for complete data set. Some reported values are estimates due to concentrations falling below method limits of detection (LOD) or quantitation (LOQ) and are clearly identified in **Table S3**.

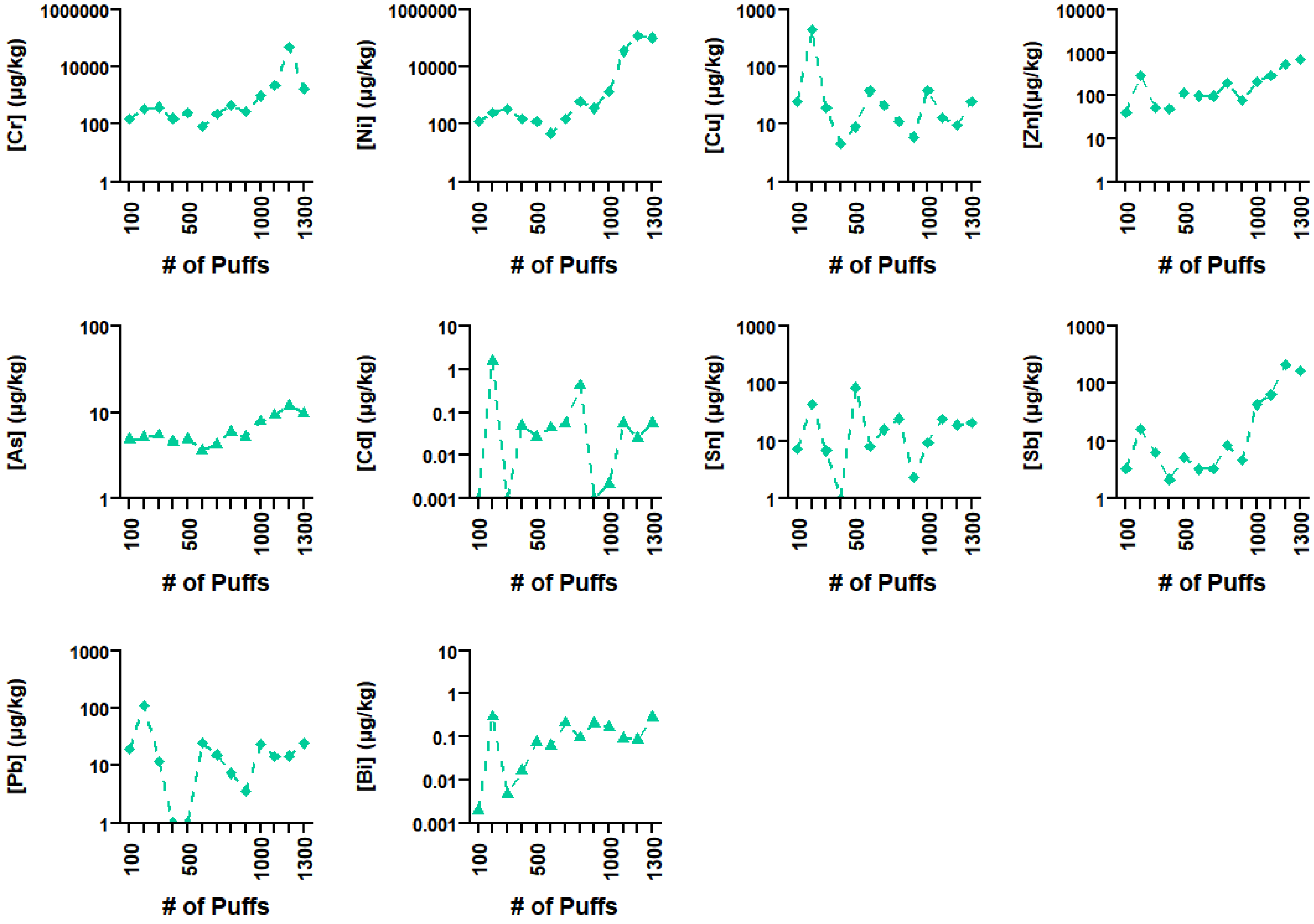

Figure S12: ELF Bar Clear concentrations (µg/kg) from full aging analysis (100-1300 puffs) (*n* = 1). See **Table S3** for complete data set. Some reported values are estimates due to concentrations falling below method limits of detection (LOD) or quantitation (LOQ) and are clearly identified in **Table S3**.

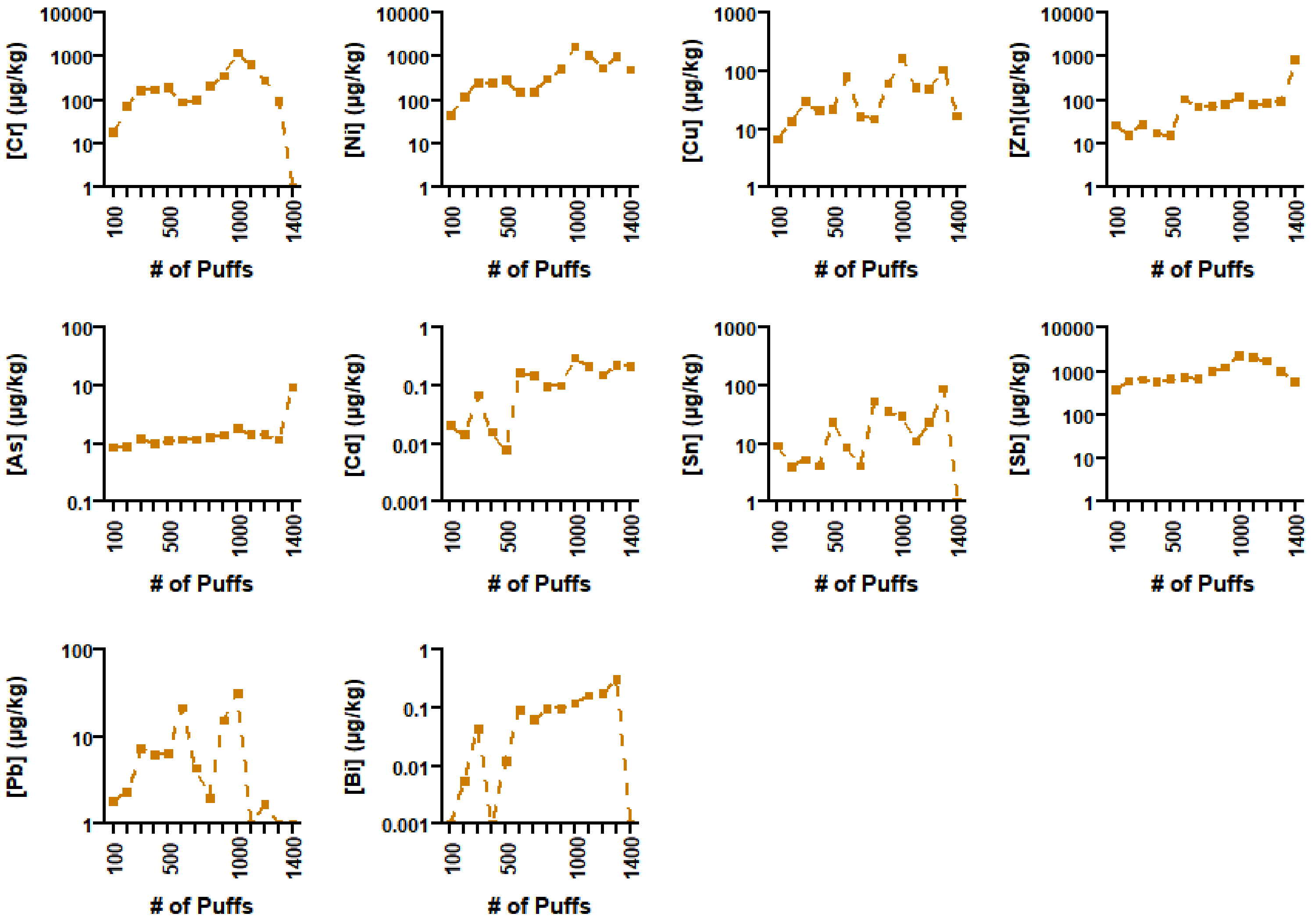

Figure S13: Flum Pebble Flavored concentrations (µg/kg) from full aging analysis (100-1500 puffs) (*n* = 1). See **Table S3** for complete data set. Some reported values are estimates due to concentrations falling below method limits of detection (LOD) or quantitation (LOQ) and are clearly identified in **Table S3**.

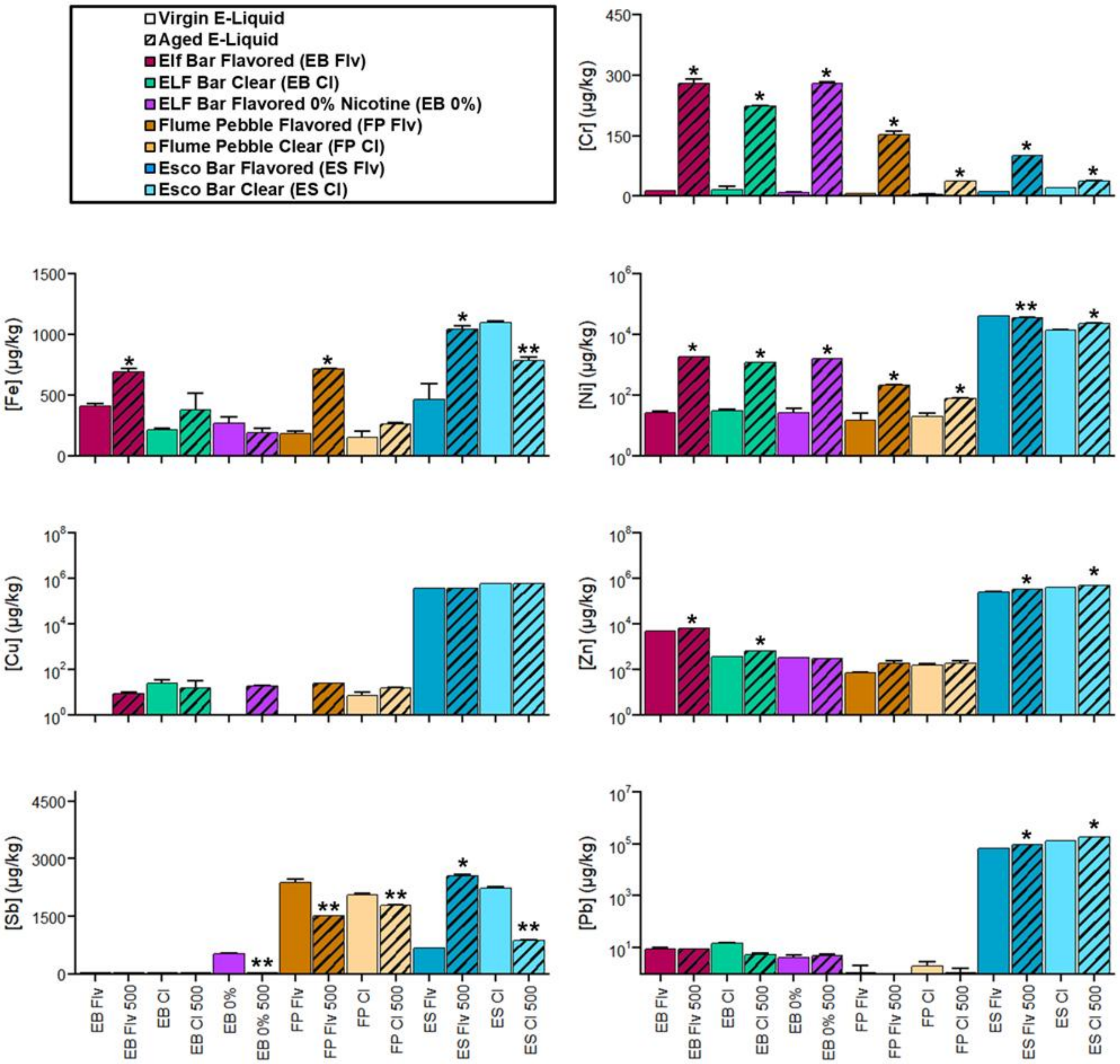

Figure S14: Virgin and 500-puff aged e-liquids were analyzed for element concentrations (µg/kg) were compared across all device types (*n* = 3) (**Table S2**). Error bars represent standard deviations. Two tailed t-tests with two sample unequal variance were performed on element concentrations of virgin and aged e-liquids to assess statistical differences in element concentrations induced by flavorings and nicotine (ELF Bar flavored and 0% nicotine) (α = 0.05) (**Table S6.1**). Single asterisks (*) indicate a significant increase in aged e-liquid concentrations. Double asterisks (**) indicate a significant decrease in aged e-liquid concentrations.

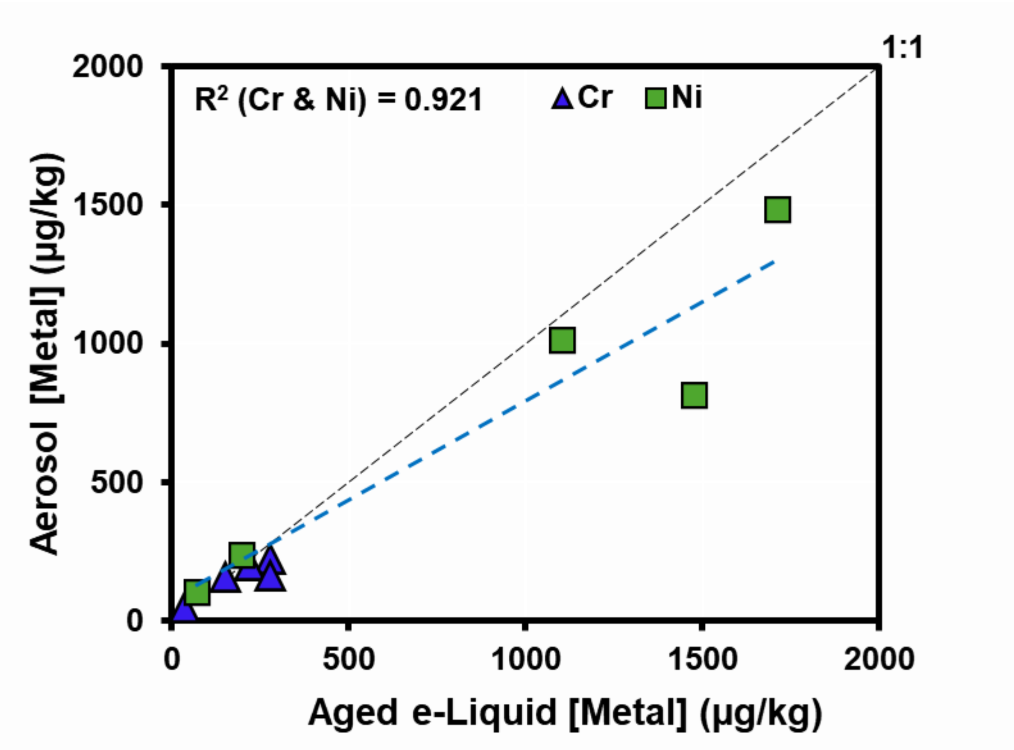

Figure S15: Cr and Ni concentrations of 500 puff aerosols (µg/kg) and 500-puff aged e-liquids (µg/kg) from ELF Bar and Flum Pebble devices plotted to assess the correlation between elemental transfer between e-liquid and aerosol (**Tables S7.1-S7.2**). Esco Bar devices were omitted from this data due to the loss of power with each device prior to 500 puffs.

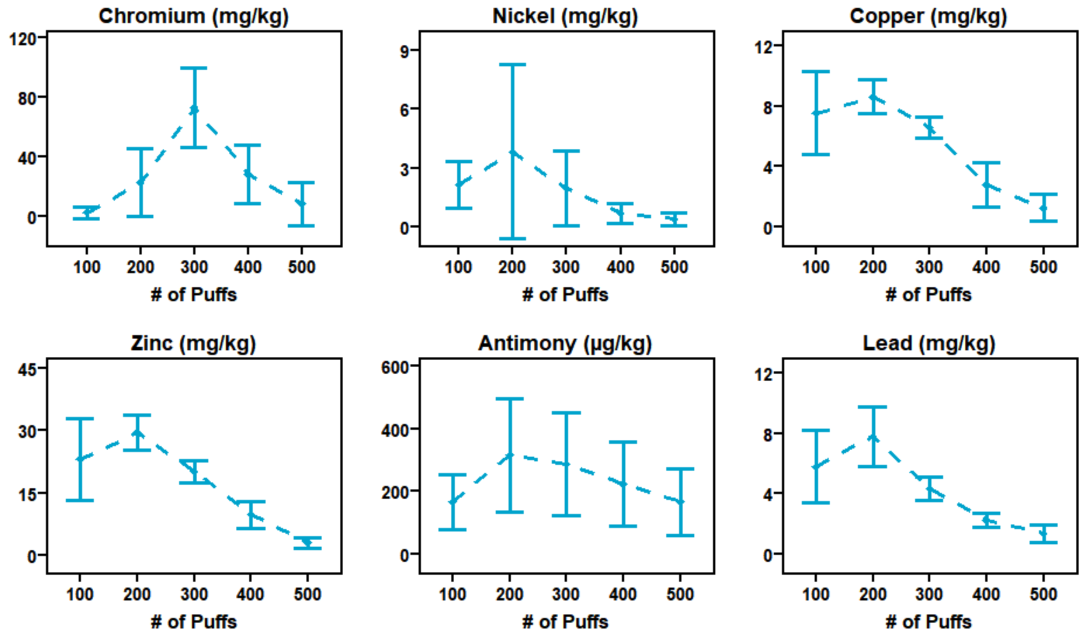

Figure S16: Esco Bar Flavored mean element concentrations (µg/kg, mg/kg) from 100-500 puffs with standard deviations (*n* = 3; **Table S3**). Some reported values are estimates due to concentrations falling below method limits of detection (LOD) or quantitation (LOQ) and are clearly identified in **Table S3**.

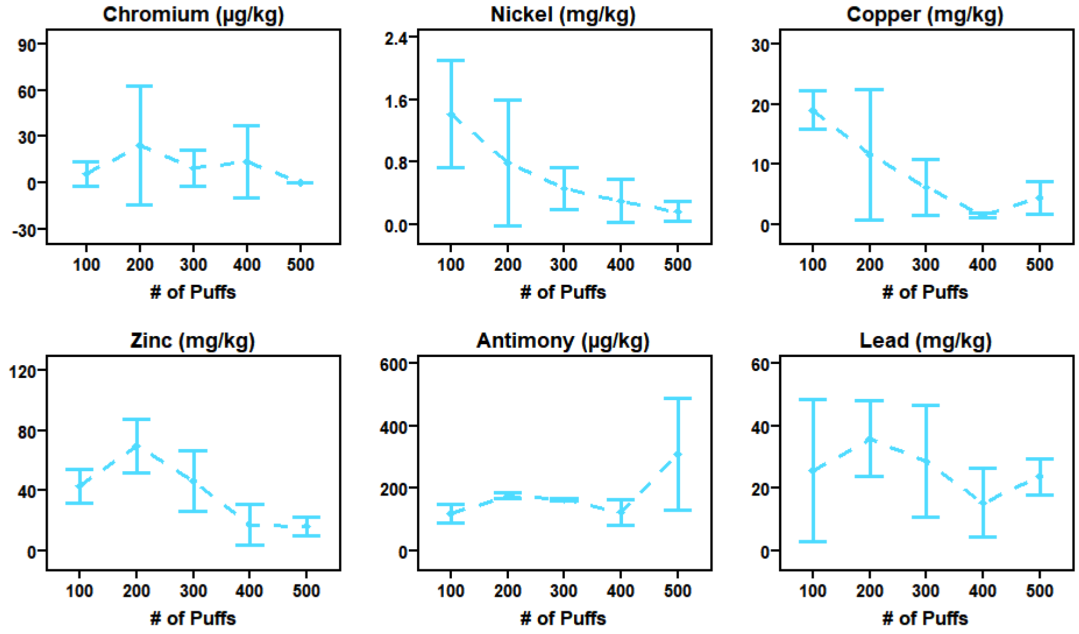

Figure S17: Esco Bar Clear mean element concentrations (µg/kg, mg/kg) from 100-500 puffs with standard deviations (*n* = 3; **Table S3**). Some reported values are estimates due to concentrations falling below method limits of detection (LOD) or quantitation (LOQ) and are clearly identified in **Table S3**.

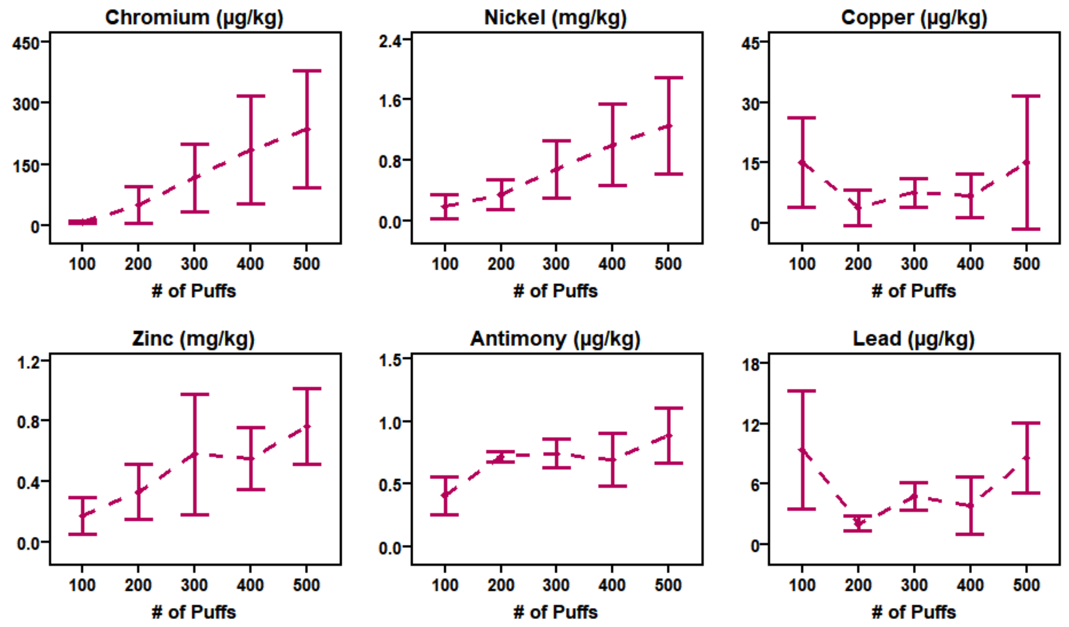

Figure S18: ELF Bar Flavored mean element concentrations (µg/kg, mg/kg) from 100-500 puffs with standard deviations (*n* = 3; **Table S3**). Some reported values are estimates due to concentrations falling below method limits of detection (LOD) or quantitation (LOQ) and are clearly identified in **Table S3**.

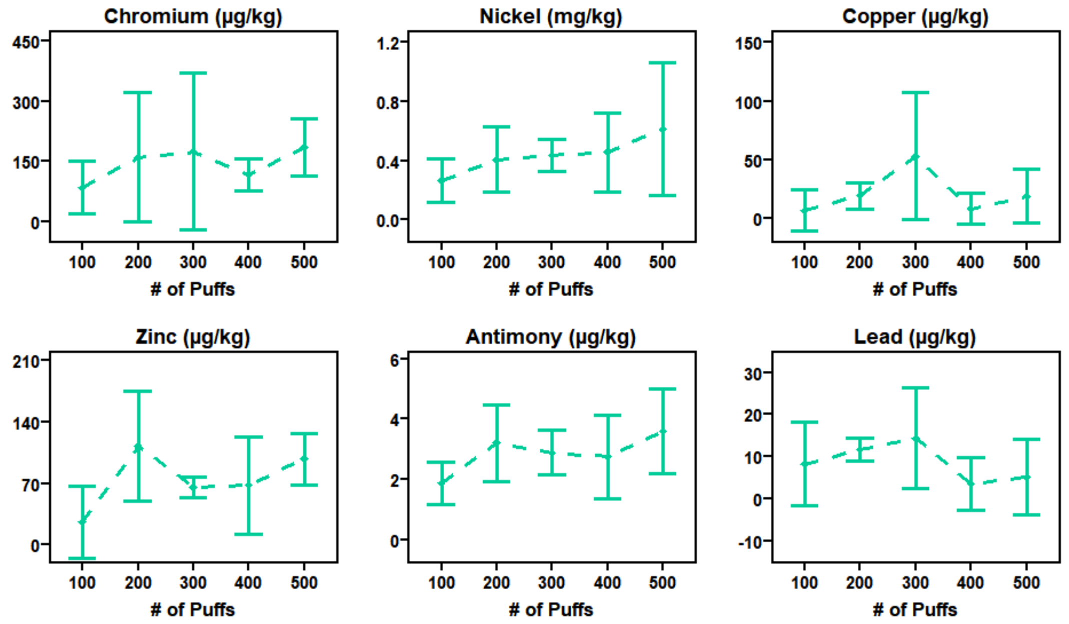

Figure S19: ELF Bar Clear mean element concentrations (µg/kg, mg/kg) from 100-500 puffs with standard deviations (*n* = 3; **Table S3**). Some reported values are estimates due to concentrations falling below method limits of detection (LOD) or quantitation (LOQ) and are clearly identified in **Table S3**.

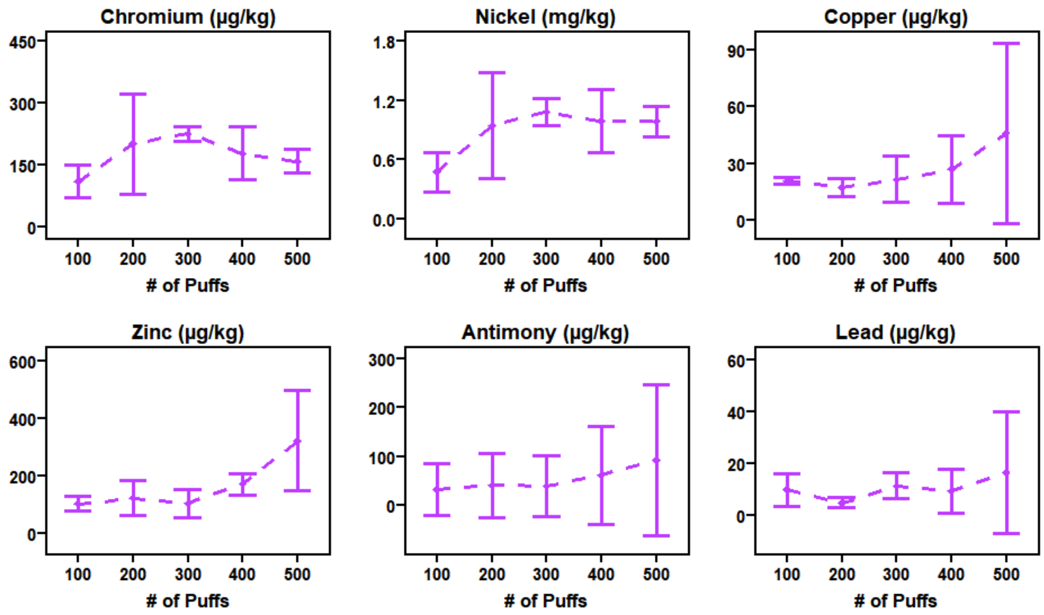

Figure S20: ELF Bar Flavored 0% Nicotine mean element concentrations (µg/kg, mg/kg) from 100-500 puffs with standard deviations (*n* = 3; **Table S3**). Some reported values are estimates due to concentrations falling below method limits of detection (LOD) or quantitation (LOQ) and are clearly identified in **Table S3**.

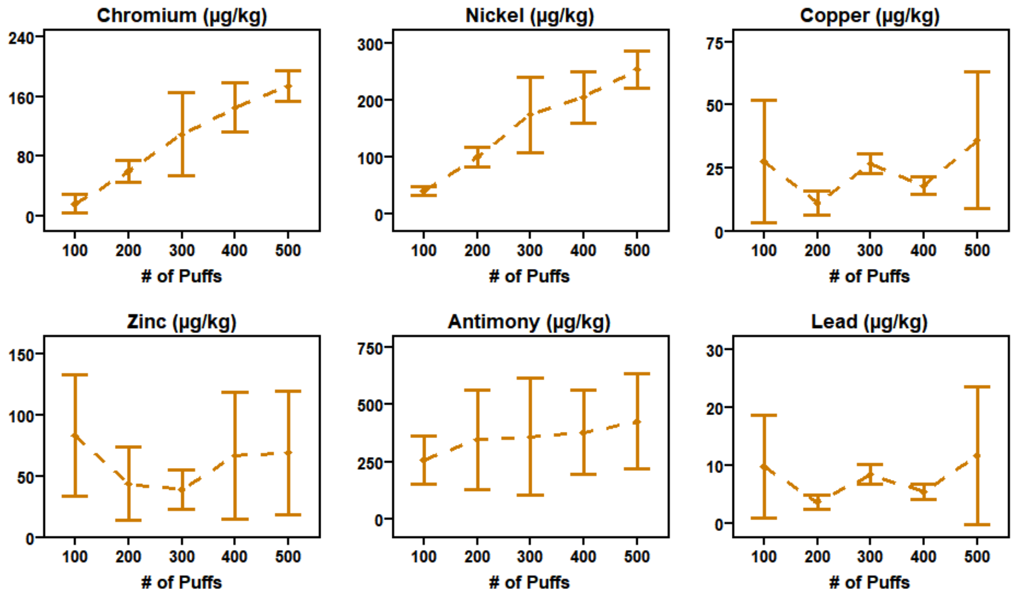

Figure S21: Flum Pebble Flavored mean element concentrations (µg/kg) from 100-500 puffs with standard deviations (*n* = 3; **Table S3**). Some reported values are estimates due to concentrations falling below method limits of detection (LOD) or quantitation (LOQ) and are clearly identified in **Table S3**.

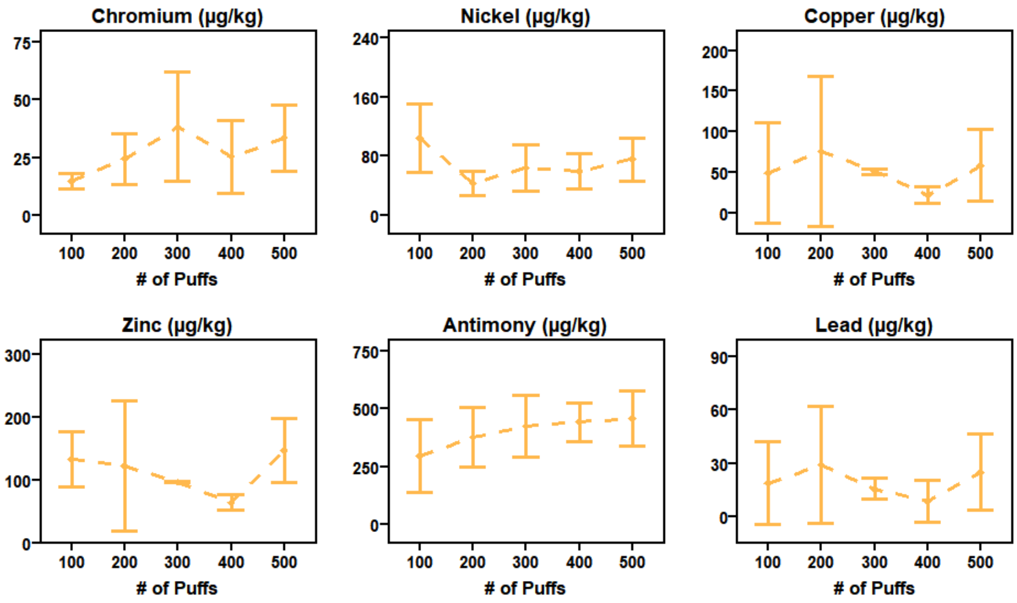

Figure S22: Flum Pebble Clear mean element concentrations (µg/kg) from 100-500 puffs with standard deviations (*n* = 3; **Table S3**). Some reported values are estimates due to concentrations falling below method limits of detection (LOD) or quantitation (LOQ) and are clearly identified in **Table S3**.

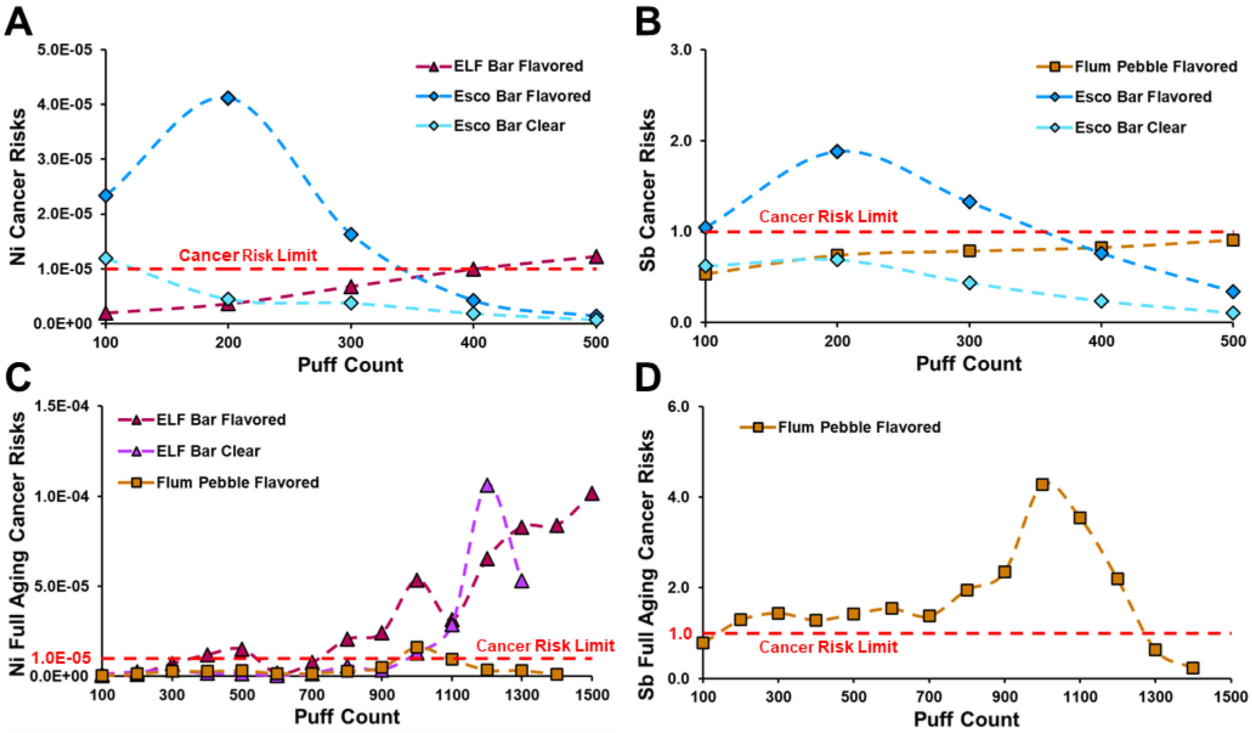

Figure S23: Cancer risk plots of Ni (100 to 500 puff means) (A), Sb (100 to 500 puff means) (B), Ni (100 to 1500 puffs; fully aged devices) (C) and Sb (100 to 1400 puffs; Flum Pebble Flavored). Cancer risk limits of 10^{-5} for Ni and 1 for Sb are shown by the red, dashed lines. Devices and elements with risk values under the risk limit omitted for clarity. Decreases in risk observed near the end of the device life cycles are reflections of reductions in aerosol generation as the devices cease to function. Users may supplement the lack of aerosol generation by puffing the devices more than the daily puff assumption (100 puffs), thus increasing exposure and risk. See **Table S16** for all tabulated data.

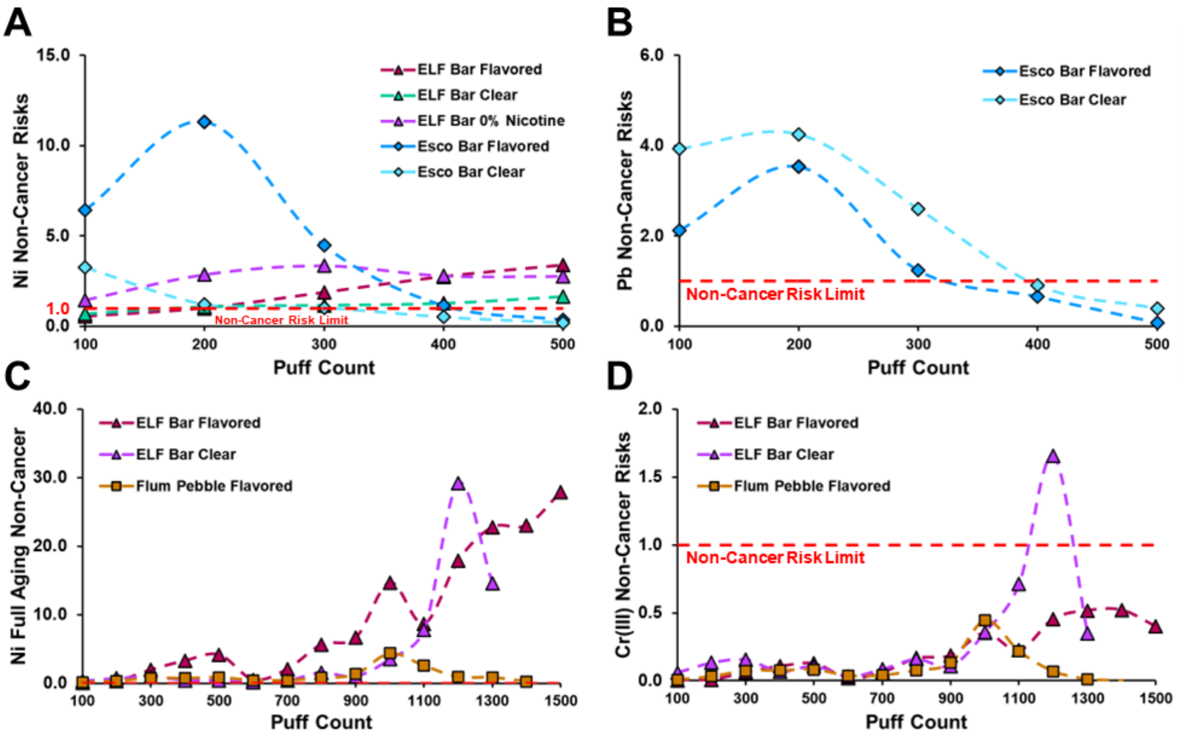

Figure S24: Non-cancer risk plots of Ni (100 to 500 puff means) (A), Pb (100 to 500 puff means) (B), Ni (100 to 1500 puffs; fully aged devices) (C) and Cr(**III**) (100 to 1500 puffs; fully aged devices). Health quotient (HQ) limits of 1 are shown by the red, dashed lines. Devices and elements with risk values under the risk limit omitted for clarity. Decreases in risk observed near the end of the device life cycles are reflections of reductions in aerosol generation as the devices cease to function. Users may supplement the lack of aerosol generation by puffing the devices more than the daily puff assumption (100 puffs), thus increasing exposure and risk. See **Table S16** for all tabulated data.

SI References

(1) Benowitz, N. L.; Iii, P. J. Daily Intake of Nicotine during Cigarette Smoking. *Clin. Pharmacol. Ther.* **1984**, *35* (4), 499–504. https://doi.org/10.1038/clpt.1984.67.
(2) Zhao, D.; Navas-Acien, A.; Ilievski, V.; Slavkovich, V.; Olmedo, P.; Adria-Mora, B.; Domingo-Relloso, A.; Aherrera, A.; Kleiman, N. J.; Rule, A. M.; Hilpert, M. Metal Concentrations in Electronic Cigarette Aerosol: Effect of Open-System and Closed-System Devices and Power Settings. *Environ. Res.* **2019**, *174*, 125–134. https://doi.org/10.1016/j.envres.2019.04.003.
(3) Aherrera, A.; Lin, J. J.; Chen, R.; Tehrani, M.; Schultze, A.; Borole, A.; Tanda, S.; Goessler, W.; Rule, A. M. Metal Concentrations in E-Cigarette Aerosol Samples: A Comparison by Device Type and Flavor. *Environ. Health Perspect.* **2023**, *131* (12), 127004. https://doi.org/10.1289/EHP11921.
(4) Olmedo, P.; Navas-Acien, A.; Hess, C.; Jarmul, S.; Rule, A. A Direct Method for E-Cigarette Aerosol Sample Collection. *Environ. Res.* **2016**, *149*, 151–156. https://doi.org/10.1016/j.envres.2016.05.008.
(5) Thomas, R. *Practical Guide to ICP-MS: A Tutorial for Beginners, Third Edition*, 0 ed.; CRC Press, 2013. https://doi.org/10.1201/b14923.
(6) *Exposure Factors Handbook. Chapter 6: Inhalation Rates*; Exposure Factors Handbook; United States Environmental Protection Agency (US EPA): Washington, D.C., 2011.
(7) Olmedo, P.; Goessler, W.; Tanda, S.; Grau-Perez, M.; Jarmul, S.; Aherrera, A.; Chen, R.; Hilpert, M.; Cohen, J. E.; Navas-Acien, A.; Rule, A. M. Metal Concentrations in E-Cigarette Liquid and Aerosol Samples: The Contribution of Metallic Coils. *Environ. Health Perspect.* **2018**, *126* (2), 027010. https://doi.org/10.1289/EHP2175.
(8) Ernstberger, H.; Neubauer, K. Chromium Speciation in Drinking Water.
(9) Zheng, J.; Iijima, A.; Furuta, N. Complexation Effect of Antimony Compounds with Citric Acid and Its Application to the Speciation of Antimony(Iii) and Antimony(v) Using HPLC-ICP-MS. *J. Anal. At. Spectrom.* **2001**, *16* (8), 812–818. https://doi.org/10.1039/b101943k.
(10) Shupack, S. I. The Chemistry of Chromium and Some Resulting Analytical Problems. *Environ. Health Perspect.* **1991**, *92*, 7–11.
(11) Zhang, Y.; O'Loughlin, E. J.; Kwon, M. J. Antimony Redox Processes in the Environment: A Critical Review of Associated Oxidants and Reductants. *J. Hazard. Mater.* **2022**, *431*, 128607. https://doi.org/10.1016/j.jhazmat.2022.128607.
(12) Agency for Toxic Substances and Disease Registry (ATSDR). *Guidance for Inhalation Exposures*; U.S. Department of Health and Human Services, Public Health Service: Atlanta, GA, 2021.
(13) Office of Environmental Health Hazard Assessment (OEHHA). *Initial Statement of Reasons: Antimony Trioxide; Proposition 65 Safe Harbors*; California Environmental Protection Agency (Cal EPA), 2022.
(14) Office of Environmental Health Hazard Assessment (OEHHA). *Final Statement of Reasons Title 27, California Code of Regulations. No Significant Risk Level: Antimony Trioxide*; California Environmental Protection Agency (Cal EPA), 2023.
(15) United States Environmental Protection Agency (US EPA). *Arsine CASRN 7784-42-1 | IRIS | US EPA*. https://iris.epa.gov/ChemicalLanding/&substance_nmbr=672 (accessed 2025-01-20).
(16) United States Environmental Protection Agency (US EPA). *Antimony trioxide CASRN 1309-64-4 | IRIS | US EPA*. https://iris.epa.gov/ChemicalLanding/&substance_nmbr=676 (accessed 2025-01-20).
(17) Agency for Toxic Substances and Disease Registry (ATSDR). *Toxicological Profile for Cadmium*; U.S. Department of Health and Human Services, Public Health Service: Atlanta, GA, 2008.
(18) Agency for Toxic Substances and Disease Registry (ATSDR). *Toxicological Profile for Chromium*; U.S. Department of Health and Human Services, Public Health Service: Atlanta, GA, 2012.
(19) California Office of Environmental Health Hazard Assessment (OEHHA). *Nickel and Nickel Compounds*. https://oehha.ca.gov/chemicals/nickel-and-nickel-compounds (accessed 2025-01-20).
(20) United States Environmental Protection Agency (US EPA). *National Ambient Air Quality Standards (NAAQS) Table*. https://www.epa.gov/criteria-air-pollutants/naaqs-table (accessed 2025-01-20).
(21) United States Environmental Protection Agency (US EPA). *Cadmium CASRN 7440-43-9 | IRIS | US EPA*. https://iris.epa.gov/ChemicalLanding/&substance_nmbr=141 (accessed 2025-01-20).
(22) California Office of Environmental Health Hazard Assessment (OEHHA). *Appendix A: Hot Spots Unit Risk and Cancer Potency Values*; Proposition 65; California Environmental Protection Agency (Cal EPA): Sacramento, CA, 2023.

(23) U.S. Food and Drug Administration (FDA). *Inhalation Reference Values in Toxicological Evaluations*; 2019.
(24) Lin, H.-C.; Buu, A.; Su, W.-C. Disposable E-Cigarettes and Associated Health Risks: An Experimental Study. *Int. J. Environ. Res. Public. Health* **2022**, *19* (17), 10633. https://doi.org/10.3390/ijerph191710633.
(25) Fowles, J.; Barreau, T.; Wu, N. Cancer and Non-Cancer Risk Concerns from Metals in Electronic Cigarette Liquids and Aerosols. *Int. J. Environ. Res. Public. Health* **2020**, *17* (6), 2146. https://doi.org/10.3390/ijerph17062146.
(26) United States Environmental Protection Agency (US EPA). *IRIS Assessments | IRIS | US EPA*. https://iris.epa.gov/AtoZ/?list_type=alpha (accessed 2024-07-28).
(27) Agency for Toxic Substances and Disease Registry. Public Health Assessment Guidance Manual (PHAGM). *ATSDR* **2022**.
(28) Rastian, B.; Wilbur, C.; Curtis, D. B. Transfer of Metals to the Aerosol Generated by an Electronic Cigarette: Influence of Number of Puffs and Power. *Int. J. Environ. Res. Public. Health* **2022**, *19* (15), 9334. https://doi.org/10.3390/ijerph19159334.
(29) Olmedo, P.; Goessler, W.; Tanda, S.; Grau-Perez, M.; Jarmul, S.; Aherrera, A.; Chen, R.; Hilpert, M.; Cohen, J. E.; Navas-Acien, A.; Rule, A. M. Metal Concentrations in E-Cigarette Liquid and Aerosol Samples: The Contribution of Metallic Coils. *Environ. Health Perspect.* **2018**, *126* (2), 027010. https://doi.org/10.1289/EHP2175.
(30) Ashraf, M. W. Levels of Heavy Metals in Popular Cigarette Brands and Exposure to These Metals via Smoking. *Sci. World J.* **2012**, *2012*, 1–5. https://doi.org/10.1100/2012/729430.
(31) Pappas, R. S.; Fresquez, M. R.; Martone, N.; Watson, C. H. Toxic Metal Concentrations in Mainstream Smoke from Cigarettes Available in the USA. *J. Anal. Toxicol.* **2014**, *38* (4), 204–211. https://doi.org/10.1093/jat/bku013.
(32) Kapiamba, K. F.; Owusu, S. Y.; Wu, Y.; Huang, Y.-W.; Jiang, Y.; Wang, Y. Examining the Oxidation States of Metals in Aerosols Emitted by Electronic Cigarettes. *Chem. Res. Toxicol.* **2024**, *37* (7), 1113–1120. https://doi.org/10.1021/acs.chemrestox.4c00033.
(33) Agency for Toxic Substances and Disease Registry (ATSDR). *Toxicological Profile for Antimony and Compounds*; U.S. Department of Health and Human Services, Public Health Service: Atlanta, GA, 2019.
(34) *Cobalt, Antimony Compounds, and Weapons-Grade Tungsten Alloy*; IARC Working Group on the Identification of Carcinogenic Hazards to Humans, Ed.; IARC monographs on the identification of carcinogenic hazards to humans; International Agency for Research on Cancer: Lyon, France, 2023; Vol. 131.
(35) National Toxicology Program (NTP). *NTP Technical Report on the Toxicology and Carcinogenesis Studies of Antimony Trioxide (CASRN 1309-64-4) in Wistar Han [Crl:WI(Han)] Rats and B6C3F1/N Mice (Inhalation Studies)*; 590; 2017; p 590.
(36) Agency for Toxic Substances and Disease Registry (ATSDR). *Toxicological Profile for Lead*; U.S. Department of Health and Human Services, Public Health Service: Atlanta, GA, 2020.
(37) Vincent, J. B. New Evidence against Chromium as an Essential Trace Element. *J. Nutr.* **2017**, *147* (12), 2212–2219. https://doi.org/10.3945/jn.117.255901.
(38) Di Bona, K. R.; Love, S.; Rhodes, N. R.; McAdory, D.; Sinha, S. H.; Kern, N.; Kent, J.; Strickland, J.; Wilson, A.; Beaird, J.; Ramage, J.; Rasco, J. F.; Vincent, J. B. Chromium Is Not an Essential Trace Element for Mammals: Effects of a "Low-Chromium" Diet. *JBIC J. Biol. Inorg. Chem.* **2011**, *16* (3), 381–390. https://doi.org/10.1007/s00775-010-0734-y.
(39) Zoroddu, M. A.; Aaseth, J.; Crisponi, G.; Medici, S.; Peana, M.; Nurchi, V. M. The Essential Metals for Humans: A Brief Overview. *J. Inorg. Biochem.* **2019**, *195*, 120–129. https://doi.org/10.1016/j.jinorgbio.2019.03.013.
(40) *Elf Bar finds an easy way around US vape import ban: a name change*. AP News. https://apnews.com/article/ecigarettes-elf-bar-fda-disposable-vaping-5245aed253ca9cdcf119483bd9cee1f1 (accessed 2025-05-08).
(41) *FDA warns stores to stop selling Elf Bar, the top disposable e-cigarette in the US*. AP News. https://apnews.com/article/ecigarettes-elf-bar-vapes-4353becf747846b528ec2aea609ed2f9 (accessed 2025-05-08).
